# Queuine links translational control in eukaryotes to a micronutrient from bacteria

**DOI:** 10.1093/nar/gkz063

**Published:** 2019-02-01

**Authors:** Martin Müller, Carine Legrand, Francesca Tuorto, Vincent P Kelly, Yaser Atlasi, Frank Lyko, Ann E Ehrenhofer-Murray

**Affiliations:** 1 Institut für Biologie, Molekulare Zellbiologie, Humboldt-Universität zu Berlin, Berlin, Germany; 2 Division of Epigenetics, DKFZ-ZMBH Alliance, German Cancer Research Center, Heidelberg, Germany; 3 School of Biochemistry & Immunology, Trinity Biomedical Sciences Institute, 152–160 Pearse Street, Trinity College Dublin, Dublin, Ireland; 4 Department of Molecular Biology, Faculty of Science, Radboud Institute for Molecular Life Sciences, Radboud University Nijmegen, Nijmegen, Netherlands

## Abstract

In eukaryotes, the wobble position of tRNA with a GUN anticodon is modified to the 7-deaza-guanosine derivative queuosine (Q34), but the original source of Q is bacterial, since Q is synthesized by eubacteria and salvaged by eukaryotes for incorporation into tRNA. Q34 modification stimulates Dnmt2/Pmt1-dependent C38 methylation (m^5^C38) in the tRNA^Asp^ anticodon loop in *Schizosaccharomyces pombe*. Here, we show by ribosome profiling in *S. pombe* that Q modification enhances the translational speed of the C-ending codons for aspartate (GAC) and histidine (CAC) and reduces that of U-ending codons for asparagine (AAU) and tyrosine (UAU), thus equilibrating the genome-wide translation of synonymous Q codons. Furthermore, Q prevents translation errors by suppressing second-position misreading of the glycine codon GGC, but not of wobble misreading. The absence of Q causes reduced translation of mRNAs involved in mitochondrial functions, and accordingly, lack of Q modification causes a mitochondrial defect in *S. pombe*. We also show that Q-dependent stimulation of Dnmt2 is conserved in mice. Our findings reveal a direct mechanism for the regulation of translational speed and fidelity in eukaryotes by a nutrient originating from bacteria.

## INTRODUCTION

Fine-tuning of protein translation is crucial for rapid, accurate and efficient production of proteins. During translation elongation, each codon in an mRNA is decoded in the acceptor (A–) site of the ribosome via an interaction between the codon and the anticodon of the matching tRNA. The optimization of this process is influenced by many factors, including the abundance of tRNAs, the usage of synonymous codons, the strength of the codon–anticodon interaction and interactions between the ribosome and the codon–anticodon complex (1,2). Synonymous codons specify the insertion of the same amino acid, but differ in their decoding properties, usage within the genome and the abundance of the tRNAs that decode them (3). Some codons require wobble base-pairing (i.e. a non-Watson–Crick interaction) between the third position in the codon and the first position of the tRNA anticodon (position 34), thus allowing one tRNA to decode two or more codons (1). The harmonization of translation rates within a proteome has been hypothesized to require that the interaction energy between codons and anticodons be relatively uniform across the genetic code. Accordingly, a novel representation of the genetic code has been proposed, which takes energy-driven as well as structural aspects of the codon–anticodon interaction into consideration. This view classifies the codons into ‘strong’, ‘weak’ and ‘intermediate’-strength based on the G/C versus A/U content of the first two codon positions into codons (4). Of note, the C- and U-ending codons differ with respect to their interaction energy, thus necessitating mechanisms that regulate their translational efficiency (5).

One possibility to modulate codon–anticodon interaction is provided by chemical modifications on the tRNAs, most prominently at the wobble tRNA nucleotide (6), and ‘weak’ codons rely more strongly on such modifications than stronger codons (4). One of the most complex RNA modifications known so far is the nucleotide queuosine (Q), which is found at position 34 of selected tRNAs (the respective base is termed queuine, q) (7,8). Queuine is a 7-deaza-guanine derivative, to which an amino-methyl side chain and a cyclopentenediol moiety are attached (9). Queuosine-containing tRNAs are produced in eubacteria in a complex biosynthetic pathway (reviewed in (10)). Interestingly, Q-containing tRNAs are also abundant in most eukaryotes, even though they do not possess the enzymes for Q synthesis. Rather, eukaryotes obtain Q through the digestion of tRNAs from nutritional sources and from gut microbes, and Q hence has been termed a micronutrient. The q base is subsequently used by the eukaryotic tRNA guanine transglycosylase (eTGT) (11) to exchange G34 for Q34 in tRNAs with a GUN/ QUN anticodon, namely tRNA^Asp^, tRNA^Tyr^, tRNA^His^ and tRNA^Asn^ (8). The respective amino acids are all encoded by two synonymous codons that carry C or U at the wobble position, but in most organisms are decoded by a single tRNA species that has G/Q34.

While Q has been known as a tRNA modification for many years, surprisingly little is known about how it affects translation *in vivo* (reviewed in (12)). In a first approximation, Q has the same base pairing properties as G to C (Watson–Crick) and U (wobble base pairing). However, *in vitro* measurement of anticodon–anticodon complexes showed that Q•U pairing displayed an approx. 3-fold increased stability compared to the G•U pair, whereas the pairing to C was destabilized by Q (5), which has led to the suggestion that Q-tRNAs enhance the translation of U-ending codons. Indeed, we recently showed in human cells that Q depletion causes reduced translational speed at the Q codons, with the U-ending codons for Asn, His and Tyr more severely slowed down than the respective C-ending codons (13). This is in line with an early study, which observed that while the unmodified G-tRNA^His^ had a preference for the CAC over the CAU codon of a heterologous gene in Xenopus oocytes, Q-tRNA^His^ showed equal decoding of the two codons (14), arguing that Q34 serves to compensate a possible disadvantage of the G – U wobble pairing.

Contrary to this, a study of codon usage across 12 *Drosophila* species showed an evolutionary selection for C-ending relative to U-ending Q codons at conserved positions in proteins, which was argued to reflect effects of Q on translational accuracy (15). Thus, another possible function of Q modification is to prevent misreading of non-cognate codons and hence to improve translational fidelity. Interestingly, Q modification shows complex, context-dependent effects on the suppression of error rates in bacteria (16).

We found earlier that Q modification shows an unexpected cross-talk with Dnmt2-dependent methylation of C38 in tRNA^Asp^ in the fission yeast *Schizosaccharomyces pombe* (17). Enzymes of the Dnmt2 family are tRNA methyltransferases that methylate position 5 of cytosine to create 5-methyl-cytosine (m^5^C) at C38 in the anticodon loop of tRNA^Asp^ (and tRNA^Gly^ and tRNA^Val^ in some organisms) (18). Our work on the Dnmt2 homolog Pmt1 from *S. pombe* (19) revealed that tRNA^Asp^ C38 methylation *in vivo* is stimulated from approx. 15% to 100% when Q is incorporated into the tRNA by eTGT (17). We have recently shown that this Q-dependence of the Dnmt2 enzyme extends to human and mice, where obtaining a Q-deficient state requires complex experimental setups and thus has previously gone unnoticed (20). Specifically, Q depletion in HeLa cells reduced the m^5^C38 levels in tRNA^Asp^ from 96 to 57%, and axenic mice fed on a Q-free synthetic diet showed reduced m^5^C38 levels (13).

In the present study, we sought to determine the generality of the effect of tRNA-Q modification on translation in eukaryotes. We used ribosome profiling to investigate the effects of Q-modification and Dnmt2-dependent m^5^C38 methylation on translation in *S. pombe*. Interestingly, Q modification showed a context-dependent effect on translational speed in that it enhanced the rate of the C-ending codons CAC (histidine, His) and GAC (aspartic acid, Asp), but slowed the rate of the U-ending codons UAU (tyrosine, Tyr) and AAU (asparagine, Asn), thus displaying a differential effect on weak (Tyr, Asn) and intermediate-strength (His, Asp) codons (4). Also, Q modification suppressed misreading of the near-cognate GGC glycine, but not the synonymous GGU codon, suggesting that the Q modification weakens base-pairing at the wobble position and thus prevents translational errors. Modification by Q resulted in reduced translation of genes with mitochondrial function; accordingly, lack of Q modification caused a respiratory defect in *S. pombe*. Finally, we found Dnmt2-dependent m^5^C38 methylation in mouse embryonic stem cells to be stimulated by Q modification of tRNA, and m^5^C38 levels were reduced in tissues from mice lacking eTGT. Altogether, our data reveal a role for tRNA queuosinylation in equilibrating translational speed and accuracy across the *S. pombe* genome.

## MATERIALS AND METHODS

### *S. pombe* strains and growth conditions

The *S. pombe* strains used in this study are shown in Supplementary Table S1. They were cultured in standard full medium (YES, 5 g/l yeast extract, 30 g/l glucose, 250 mg/l adenine, 250 mg/l histidine, 250 mg/l leucine, 250 mg/l uracil, 250 mg/l lysine) or minimal medium (EMM) with 2% glucose (to select for plasmids for *pmt1*^+^ overexpression or the control vector). For growth under respiratory conditions, 0.5% (w/v) yeast extract with 3% (w/v) glycerol as a carbon source was used. Synthetic queuine, kindly provided by Hans-Dieter Gerber and Gerhard Klebe (Universität Marburg) (21), was added to 0.03 μM as indicated. Replacement of chromosomal *pmt1*^+^ by *pmt1-S80P* was constructed by CRISPR-Cas9 genome editing (22).

Growth curves of fission yeast cultures were obtained using a microplate reader (SynergyH1, BioTek). 100 μl cultures in a 96-well plate were inoculated to an optical density at 600 nm (OD_600_) of 0.1 in supplemented EMM with or without 0.03 μM queuine. OD_600_ was measured in 10 min intervals with double orbital shaking.

### Cultivation of mouse embryonic stem cells

E14Tg2a ESCs were maintained on gelatin coated-dishes without feeder cells. ESCs were cultured in serum free medium containing NDiff 227 (StemCells, Inc.) supplemented with MEK inhibitor PD0325901 (1 μM), GSK3 inhibitor CHIR99021 (3 μM) and LIF (1000 U/ml, Milipore). Queuine treatment was performed using 2i-medium supplemented with 0.05 or 0.5 μM of queuine. Cells were treated for 1, 2 or 3 days and harvested for RNA extraction.

### Animals

B6.129S4-Qtrt1Gt/+ mice (N6F3) (23) were bred in specific pathogen-free conditions at the Transgenic Facility of Trinity College Dublin. Brain and liver samples were recovered from adult animals of the respective genotypes at 12–16 weeks of age and stored in RNAlater^®^ (Thermo Fisher Scientific) before extraction of RNA.

### Ribosome profiling

Ribosome footprinting was performed essentially as described (24,25). *Schizosaccharomyces pombe* cultures were grown at 30°C to an OD_600_ of 1.0, 150 μg/ml cycloheximide was added to the medium and cells were incubated for further 15 min at 30°C. Cells were harvested and flash-frozen immediately in liquid nitrogen. Fifteen OD of each sample was lysed by adding 100 μl lysis buffer (20 mM Tris–HCl pH 8.0, 150 mM NaCl, 5 mM MgCl_2_, 1% Triton X-100, 200 μg/ml cycloheximide, 1% triton, protease inhibitor) and 1 g chilled glass beads (0.5 mm diameter). Samples were homogenized using a FastPrep-FP120 (MP Biomedicals) at level 6 for 13 s and subsequently diluted with 400 μl lysis buffer. Two centrifugation steps (1: 5 min, 4°C, 16 000 × g; 2: 15 min, 4°C, 16 000 × g) removed glass beads and cellular debris, respectively. In order to assess sample quality, polysome profiles were analyzed before footprint isolation. For this purpose, 400 μl of the supernatant were applied to linear 17.5 to a 50% sucrose gradient in 15 mM Tris–HCl pH 8.0, 15 mM MgCl_2_, 300 mM NaCl. Centrifugation was carried out at 35 000 rpm for 2.5 h at 4°C in a Beckmann SW60 rotor. Gradients were eluted with an ISCO UA-6 gradient fractionator, and polysome profiles were recorded by continuously monitoring the absorbance at 254 nm. For footprint isolation, 400 μl of the supernatants were digested with 4 U DNase I (ThermoScientific) and 800 U of RNase I (Ambion), for 45 min at room temperature with gentle shaking. 800 U of RNasin ribonuclease inhibitor (Promega) was added to quench the reaction. The samples were run on a 17.5–50% sucrose gradient to isolate monosomes. Footprint monosome RNAs were end-repaired with T4 polynucleotide kinase (TaKaRa), and size selected (28–31 nucleotides) on a 15% polyacrylamide Tris-borate–EDTA–urea gel. Sequence libraries from ribosome footprints and total *S. pombe* RNA were prepared according the manufacturer's protocol using NEB NEXT Small RNA library Prep Set for Illumina (Multiplex Compatible) E7330 (26). For details and quality control, see Supplementary Information.

### Translational error assay

Reporter plasmids to assay the frequency of translational errors *in vivo* were constructed based on plasmids developed to measure error rates in *Escherichia coli* (16). Plasmids (derivatives of pSLF173, see Supplementary Table S2) carrying *E. coli* β-galactosidase expressed under control of the *S. pombe nmt1* promoter were constructed that carry point mutations in codons encoding the active site amino acids aspartic acid 201 (D201) or glutamic acid 537 (E537) by amplifying the respective lacZ versions using primers XhoI_LacZ_pJC27.fw and LacZ_pJC27_BglII.rev (Supplementary Table S3) and cloning the resulting fragments into pSLF173 with XhoI/ BglII (all constructs were verified by sequence analysis). D201 (Asp), normally encoded by GAC or GAU, was mutated to Glu (GAA or GAG), or to Gly (GGU or GGC). E537(Glu) was mutated to Asp (GAC or GAU). Furthermore, Y503 was mutated to Cys (UGC or UGU). Mistranslation of the mutated codons D201 and E537 by tRNA^Asp^ or tRNA^Glu^, respectively, will restore activity of the reporter and thereby indicate translational error rates *in vivo. S. pombe* strains carrying these plasmids were cultured in minimal medium with or without 0.03 μM queuine. ß-Galactosidase activity was assayed using ortho-nitrophenyl-ß-d-galactopyranoside (ONPG) as described (27). Briefly, 1 ml of a yeast overnight culture was harvested and resuspended in 500 μl Z-buffer. After adding 50 μl of 0.1% SDS and 50 μl chloroform, cells were lysed by vigorous vortexing. 100 μl of ONPG (4 mg/ml) was added and the reaction incubated for 30 min at 37°C. Reaction was quenched with 500 μl 1M Na_2_CO_3_, and the optical density at 420 nm (OD_420_) was measured. Since strains expressing the wild-type ß-galactosidase had much higher activity, only 50 μl of the respective cultures were harvested and assayed for only 2 min. ß-Galactosidase activity was calculated using the following formula, where *V* is the harvested volume and t the incubation time: Activity = 1000 × OD_420_/*V* × *t* × OD_600_.

### High-throughput bisulfite sequencing of tRNA^Asp^

Total RNA was isolated from mouse brain and liver by placing 200 mg of the tissue in a 2 ml Eppendorf tube together with eight steel beads (2 mm). Tubes were flash frozen in liquid nitrogen and homogenized in Mixer Mill 400 (Retsch, Germany). RNA was extracted from the resulting powder with 1 ml TRIzol (Invitrogen) following the manufacturers instructions. For RNA extraction from ESCs, cells were lysed directly in TRizol, and RNA extraction was performed according to manufacturers instructions. Bisulfite sequencing of tRNAs was performed as described previously (17,28). Primer sequences are listed in Supplementary Table S3. Processing included trimming of PCR primers, selection of high quality reads and sorting of the reads based on the sequence in the degenerate region of the RT-primer. Processed reads were analyzed for bisulfite conversion using BiQ Analyzer HT (29).

### High-throughput sequencing of RNA (RNA-Seq)

After quantification of total RNA and quality assessment on an Advanced Analytical Fragment Analyzer using the DNF-472 High Sensitivity RNA Analysis Kit (all Advanced Analytical Tech. Inc; Ankeny Iowa, USA), the mRNA was isolated from high quality total RNA extracts by using the NEBNext Poly(A) mRNA Magnetic Isolation Module (E7490). Illumina-compatible RNA-libraries were constructed by using the NEBNext Ultra RNALibrary Prep Kit for Illumina (E7530) as well as the NEBNext Multiplex Oligos for Illumina (E7335) following the distributors instruction manuals. Multiplexed RNA libraries were mixed in equilibrium according to their Qubit (Life Technologies) measurements, fragment distribution profiles on the Advanced Anayltical Fragment Analyzer and quantification of library 5′-and 3′- ends by Real Time PCR. For each library, 30 Mio clusters were sequenced in a 1 × 75 base mode on a Illumina NextSeq500 instrument according to the manufacturer's protocol and using the NextSeq 500/550 High Output Sequencing Kit v2 (75 cycles) (Product Code: FC-404-2005).

### Ribosome footprint and RNA sequencing analysis

Ribosome footprinting analysis was conducted mainly as previously described (26,30,31). Sequences from ribosome footprints were trimmed (quality score ≥ 30), cleansed of adaptor sequences (AGATCGGAAGAGCACACGTCT), and size-selected (25–35nt). Remaining reads were aligned to rRNA and tRNA (main and mitochondrial) sequences from arb-silva (https://www.arb-silva.de/), Pombase (https://www.pombase.org/) and gtRNA (http://gtrnadb.ucsc.edu/), using Bowtie (seed length 23nt, up to two mismatches with base quality ≥70). Mapped reads were discarded, and unmapped reads were further aligned to transcripts sequences from Pombase (cDNA devoid of dubious sequences). The total number of reads for each replicate was between 2 844 504 and 27 469 062, among which 857 876 to 11 543 820 reads mapped to mRNA transcripts. The latter reads were length-stratified between 25 and 32nt. We examined the counts of each read start occurring at 20nt up- or downstream of the start codon to determine which lengths exhibited 3nt periodicity, sufficient read amounts and a clear peak at 12nt/start codon, and were therefore suitable for further analysis. Restricting the aligned reads to these lengths, and after further basic quality controls (consistent coding sequence, as annotated in Pombase), we assigned A-site position at read start +15nt (+1nt offset to match the open reading frame wherever necessary). We calculated bulk codon occupancy by dividing the codon proportions in the A-site by the codon usage (codon proportions averaged on the 3 codons directly adjacent to the 3′ side of the A site. For single sample analysis, we used codon usage compiled from CDS sequences from the mRNAs that are represented in the footprints). Comparisons between conditions were obtained by dividing bulk codon occupancy in one condition by bulk codon occupancy in a control condition. In addition, read counts per mRNA were determined. tRNA adaptation indices, denoted tAI, were calculated for each codon from formulas 1 and 2 in (32). Codon-anticodon pairing efficiencies were taken from (33), and tRNA counts by anticodon for *S. pombe* were retrieved from the genomic tRNA database http://gtrnadb.ucsc.edu (34). Spearman rank correlation coefficient was calculated between the vector of 1/tAI for each codon and the vector of A-site codon occupancies. Mean relative enrichment of arginine codons was calculated using a custom Python script reproducing calculations described in (35). Offsets from 90 codons upstream to 90 codons downstream of the A-site were used.

Sequences from RNA-seq were processed like ribosome footprints except size selection (reads at least 25nt long were considered). Read counts per replicate were between 23 308 777 and 78 566 554, resulting in 11 065 179 to 68 433 052 reads mapped to mRNA transcripts. The count of reads aligning to a specific mRNA was used to normalize the respective number of footprints to calculate translation efficiency. The procedures described here were implemented and carried out using Galaxy (36) and custom Python and R scripts. Differential translation efficiency was assessed using Xtail (37). Inclusion threshold was set at 0.05 (unadjusted *P*-values) or 0.1 (Benjamini–Hochberg-adjusted *P*-value, adjusted for multiple testing, for *pmt1Δ* + v/ *pmt1*^+^ OE) for use in further enrichment analysis for comparisons wt/ wt+Q, *pmt1Δ*/ *pmt1Δ*+Q, *pmt1Δ*/wt, *pmt1Δ*+Q/ wt+Q, *pmt1Δ*/ wt + Q and *pmt1Δ* + v/ *pmt1*^+^ OE. GO enrichment analysis was performed using DAVID functional annotation tool, with significance level equal to 0.1 on *P*-values adjusted for multiple testing.

### Quantification and statistical analysis

Mean and standard deviation were used in the figures, unless stated otherwise. Ribosome profiling and RNA-seq data are available in the NCBI GEO database, record GSE102376. Details on software are available in Supplementary Table S4.

### Bisulfite sequencing analysis

The tRNA cytosine-5 methylome was analysed by bisulfite sequencing as described (38). Briefly, sequenced reads were aligned using BSMAP with reference tRNA sequences downloaded from the genomic tRNA database http://gtrnadb.ucsc.edu/, and summarized so as to remove sequences which would be identical if all Cs are converted to Us. Aligned reads were quality-controlled, filtered for unconversion artefacts and ambiguous alignment. Remaining reads were identified to a Poisson distribution. Potential methylation status was derived using a Poisson test and the calculated distribution parameters.

### Immunoblotting

For the analysis of Cox2 protein expression, cells were harvested in logarithmic phase and lysed in 1.85 NaOH/7.4% ß-mercaptoethanol (39). Proteins were precipitated with 25% TCA, washed with acetone and resuspended in 5% SDS. After SDS-PAGE, proteins were transferred to nitrocellulose membranes, according to standard techniques. Membranes were incubated with 1:3000 anti-Cox2 antibody (40) followed by 1:50 000 anti-rabbit HRP antibody (Sigma, A0545), or with 1:2000 anti-tubulin antibody (Abcam, ab6161) followed by 1:50 000 anti-rat HRP antibody (Abcam, ab6734).

See Supplementary Information for further Materials and Methods.

## RESULTS

### Queuosinylation of tRNAs increases translational speed for C-ending histidine and aspartate codons and reduces speed for U-ending asparagine and tyrosine codons in *S. pombe*

Both Q34 and m^5^C38 modification of tRNAs occur in the anticodon stem-loop, raising the question whether they affect codon–anticodon interaction and thus translational speed. To investigate translational effects *in vivo*, ribosome profiling was performed with *S. pombe* cells (24,26). The individual and combined effects of Dnmt2-dependent m^5^C38 methylation and queuosinylation of tRNAs were determined for wild-type (wt) and *pmt1Δ* cells that were cultured in the presence or absence of queuine (three independent biological replicates of each condition; see Supplementary Information for quality control).

**Figure 1.**
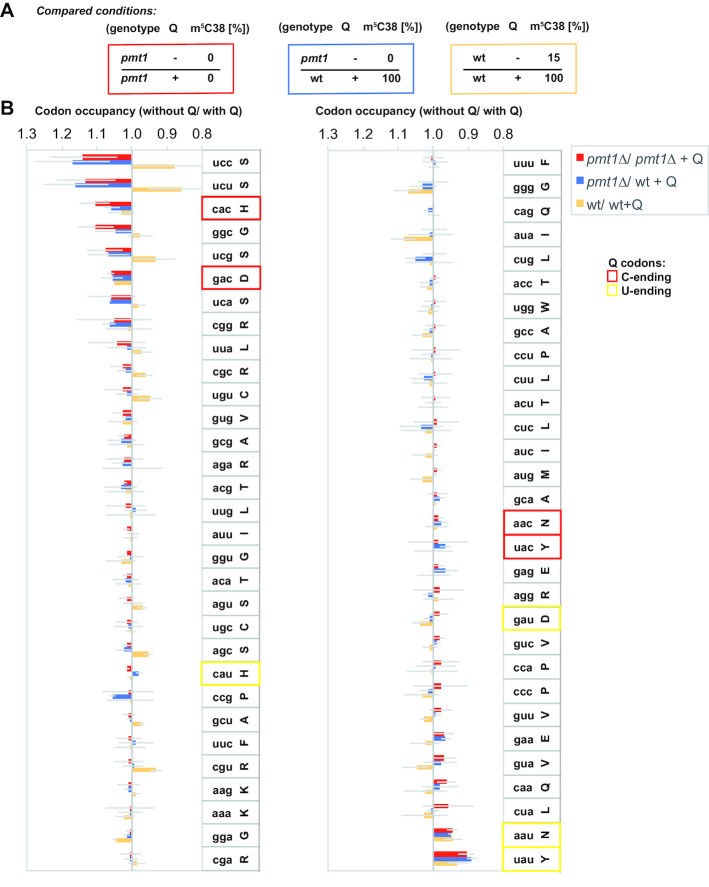
Queuosine modification of tRNAs increases the translational speed of the C-ending codons for His and Asp and decreases the speed of the U-ending Asn and Tyr codons. (**A**) Overview of the experimental conditions for ribosome profiling to determine the effect of Q modification on translation. m^5^C38 indicates the level (%) of C38 methylation of tRNA^Asp^ in a given sample. Q designates samples with (+) or without (–) Q34-modification of tRNAs. (**B**) Codon-specific changes in ribosomal A-site occupancy in the absence of Q modification in comparison to with Q (mean ± SEM, n = 3), as measured by ribosome profiling. C-ending Q codons are boxed in red, U-ending codons in yellow. Thin gray lines give the SEM.

To investigate the effect of Q modification on the translational speed of individual codons, we first compared the two conditions of no *versus* full Q modification in the absence of m^5^C38 on tRNA^Asp^, i.e. in the *pmt1Δ* strain. This was done by calculating the ratio of occupancy in *pmt1Δ* without Q to that of *pmt1Δ* grown with Q (*pmt1Δ*/ *pmt1Δ* + Q, Figure 1A and B, red bars). If the ratio is higher than 1, this indicates that the ribosome occupancy is increased in *pmt1Δ* relative to *pmt1Δ* + Q. This is interpreted to mean that translational speed is decreased in the absence of Q. Interestingly, we found two patterns in the response of the Q codons to the presence or absence of the Q modification. For the C-ending Asp and His codons (GAC and CAC), the absence of Q increased occupancy time (occupancy ratio unmodified/modified > 1) in the ribosomal A-site (Figure 1B), therefore decreasing translational speed, whereas the residency of the ribosome on the respective U-ending Asp and His codons GAU and CAU was unaffected (Figure 1B). Thus, the Q-modification of the respective tRNAs caused faster translation for the C-ending Asp and His codons relative to the U-ending codons.

A second response was observed for the Q-containing tRNAs for Asn and Tyr. In the absence of Q, U-ending codons (AAU and UAU) showed lower ribosomal occupancy (occupancy ratio unmodified/ modified < 1), and thus faster translation. The Q34 modification thus slows translation of the U-ending codon relative to the C-ending codon, whereas the C-ending codons (AAC and UAC) were unaffected by Q modification (Figure 1B). Therefore, Q modification reduces translational speed of the U-ending codons for Asn and Tyr.

**Figure 2.**
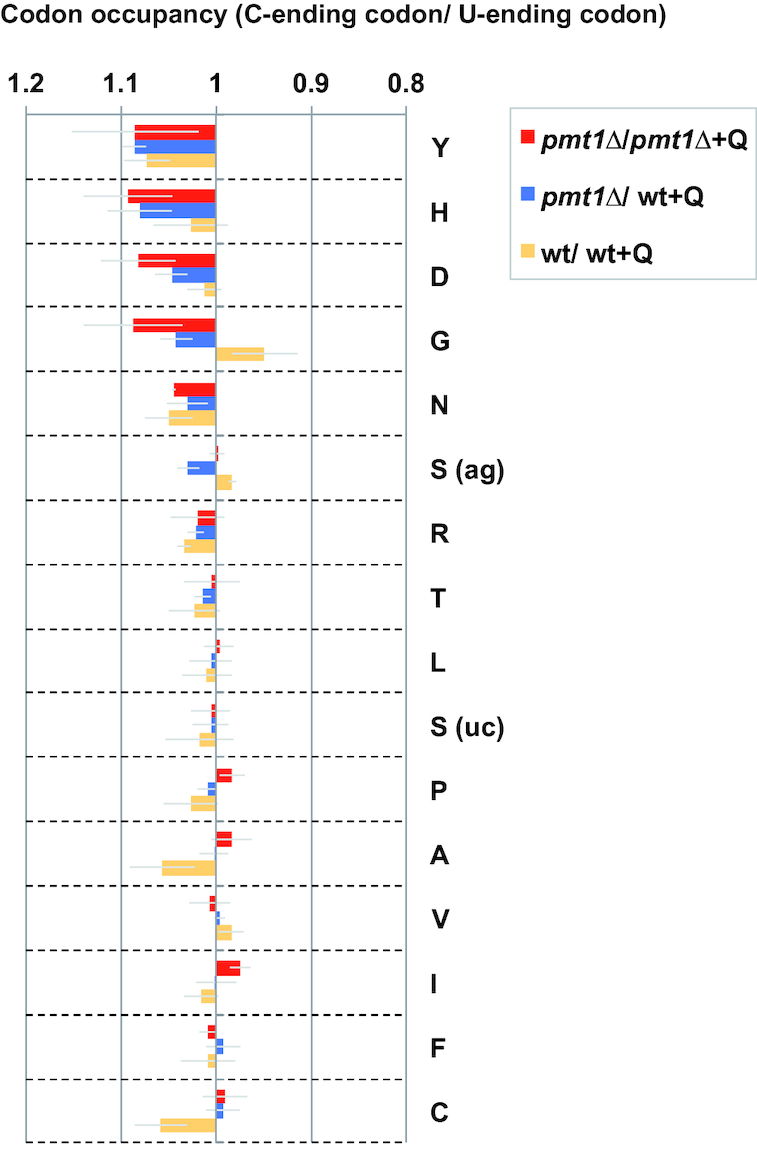
Translational speed of C-ending Q codons relative to U-ending codons was decreased in the absence of Q modification (mean ± SEM (thin gray lines), *n* = 3). Experimental conditions used are as in Figure 1.

Further analysis addressed how Q differentially affects the respective U- versus C-ending codon pairs in the absence of m^5^C38. This was determined by calculating the ratio of reduced translational speed generated at the C- relative to the U-ending codon (Figure 2, *pmt1Δ*/ *pmt1Δ* + Q, red bars). The results showed, in all four cases of Q codons, that the C-ending codon showed higher ribosome occupancy than the U-ending codon when the tRNA was not Q-modified, meaning that the C-ending codons became slower than the U-ending codons for translation in the absence of Q modification. With the exception of the glycine codons, this differential effect on C- versus U-ending codons was only slight for other codon pairs.

Codons other than those of Q-containing tRNAs also showed changes in codon occupancy. Notably, the glycine codon GGC showed higher occupancy in the presence of Q, which may relate to our observation that the GGC codon is sensitive to misreading by tRNA^Asp^ (see below). Furthermore, the four UCN serine codons displayed slower translation in the absence of Q (Figure 1B), though the differences among replicates were substantial and were not statistically significant. These effects may reflect differences in tRNA pools and tRNA availability. Indeed, we observed variability in tRNA^Ser^_AGA_ levels across replicates (Supplementary Figure S1A). We note that other ribosome profiling studies in *S. cerevisiae* observe similarly high variability among replicates and effects on seemingly unrelated codons that are on par with those on the expected codons (41).

We next considered the consequence of the combined effect of Q modification and m^5^C38 on codon occupancy by calculating the ratio of occupancy in *pmt1Δ* versus wt + Q (Figure 1A and B, blue bars). While m^5^C38 on tRNA^Asp^ is absent in *pmt1Δ*, it is present to 100% in wt + Q due to the stimulation of Dnmt2/ Pmt1 activity by Q (17). This comparison allowed us to evaluate the additional effect of m^5^C38 relative to *pmt1Δ*/ *pmt1Δ* + Q (Figure 1B, red bars). Altogether, the effects on codon occupancy were rather similar to those of *pmt1Δ*/ *pmt1Δ* + Q, and differences were not statistically significant. We conclude that the effect on translation is predominated by Q, and that m^5^C38 at most has a mild modulatory effect on codon occupancy in addition to Q modification.

As a third comparison, we investigated the change in codon occupancy in wt without Q to wt with Q (wt/wt + Q, Figure 1A and B, orange bars). This analysis is complicated by the fact that wt cells carry only partially C38-methylated tRNA^Asp^ (approx. 15%), but upon addition of Q, m^5^C38 increases to 100% (17). The ratio of codon occupancy was similar for all Q codons, with the exception of the GAU Asp codon, where the ratio was higher than 1. This suggests that changing from 15% m^5^C38 and 0% Q to 100% of m^5^C38 and Q may show a mild trend towards decreased translational speed of this codon, and this results in a loss of the preferential reduction of translational speed at the C-ending versus the U-ending Asp codon (Figure 2, orange bars compared to red and blue bars). Furthermore, the effects on non-Q codons (e.g. the serine codons) appeared reverted in wt/ wt + Q (Figures 1B and 2), though there was large variability among replicates, and the differences to the other conditions were not statistically significant.

We were interested to see whether the two types of translation responses of the Q codons described above reflected differences in the levels of Q modification of the respective tRNAs. This was not the case, since our analysis showed that tRNA^Asp^ and tRNA^Tyr^ were equally strongly Q-modified when grown in Q-containing medium, and tRNA^His^ and tRNA^Asn^ were modified to ∼90% (Supplementary Figure S1B, with some variation among replicates). Of note, the Q levels in tRNAs observed here in *S. pombe* were consistent with earlier observations in mice that tRNA^Asp^ and tRNA^Tyr^ are preferentially modified relative to tRNA^His^ and tRNA^Asn^ (20,42).

Taken together, these results indicate that one function of Q-modification in tRNAs is to improve translation of C-ending codons relative to U-ending codons in *S. pombe*. This contrasts with our recent analysis of the effect of Q in human cells, where three U-ending codons were more strongly slowed down in the absence of Q than the respective C-ending codons (13). However, our results are in line with the observation of a preference for C-ending codons across evolutionary time at conserved protein residues in Drosophilids (15).

### Dnmt2-dependent m^5^C38 methylation causes mild changes in translational speed of aspartate codons

**Figure 3.**
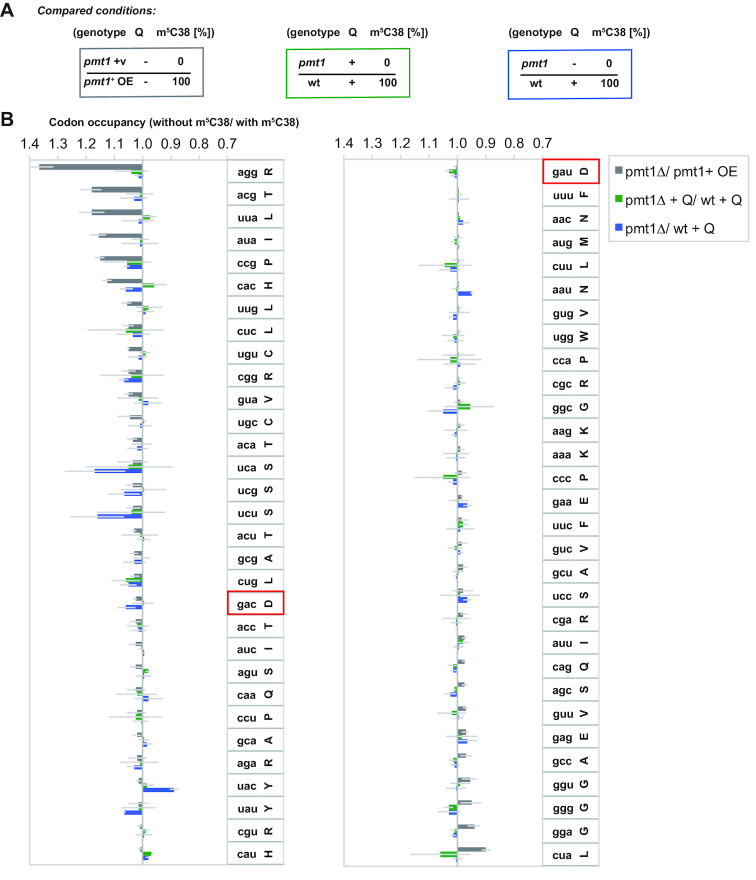
Global effect of Dnmt2/ Pmt1-dependent tRNA^Asp^ methylation on codon occupancy. Codon occupancy in samples without m^5^C38 tRNA^Asp^ methylation was compared to those with m^5^C38. (**A**) Overview of sample comparisons regarding the presence or absence of m^5^C38 and Q. Representation as in Figure 1A. Sample comparisons were *pmt1*Δ cells carrying a vector control (+v) compared to control *pmt1*^+^ OE (overexpression) cells (cells cultivated in minimal medium, gray); *pmt1*Δ + Q compared to wt +Q (green); and *pmt1*Δ compared to wt + Q (blue, same data as in Figure 1B). Values are mean ± SEM (representation as in Figure 1B). (**B**) Codon-specific changes in ribosomal A-site occupancy in the absence of m^5^C38 (*pmt1*Δ) compared to with m^5^C38 by *pmt1*^+^ overexpression (OE) or by the addition of Q. Representation as in Figure 1B.

We next sought to investigate the effect of C38 methylation of tRNA^Asp^ on translational speed. To determine the effect of quantitative m^5^C38 methylation in the absence of Q, cells over-expressing *pmt1^+^* were compared to cells carrying an empty vector. *pmt1*^+^ overexpression (OE) is the only experimental condition in which we can obtain 100% m^5^C38 methylation in the absence of Q (17), and cells had to be cultivated in minimal medium to select for the presence of the respective *pmt1*^+^ plasmid. As a control, cells carrying the empty vector and cultivated in minimal medium were used (*pmt1*Δ +v/ *pmt1*^+^ OE, Figure 3A and B, grey bars) in order to obviate differences in growth conditions. We furthermore calculated ratios of codon occupancy for the effect of m^5^C38 in the presence of Q (*pmt1*Δ + Q/wt + Q, grown in full medium, Figure 3B, green bars), or changing both m^5^C38 and Q (*pmt1*Δ/ wt + Q, grown in full medium, Figure 3B, blue bars, same data as in Figure 1B, blue bars). The Asp codon GAC showed a mild increase in ribosome occupancy in *pmt1*Δ compared to *pmt1*^+^ overexpression (Figure 3B, gray bars) and *pmt1*Δ compared to wt + Q (Figure 3B, blue bars), and the GAU codon occupancy was weakly increased by m^5^C38 in the presence of Q (Figure 3B, *pmt1*Δ + Q/ wt + Q, green bars), indicating that they were translated slightly slower when tRNA^Asp^ carried C38, rather than m^5^C38.

Notably, several codons displayed stronger Dnmt2-dependent changes in ribosome occupancy than the Asp codons. Most prominently, the arginine (AGG), threonine (ACG) and leucine (UUA) codons showed increased occupancy in the absence of m^5^C38 in the *pmt1*Δ/ *pmt1*^+^ OE comparison (Figure 3B, gray bars), but not the other comparisons. This difference may be the result of the necessity to cultivate cells for the *pmt1*Δ/ *pmt1*^+^ OE comparison in minimal medium, which also precluded us from comparing the effect of *pmt1*^+^ overexpression in the absence of Q (100% m^5^C38, 0% Q) to that in wt + Q (100% m^5^C38, 100% Q) due to large differences of cultivation conditions (full versus minimal medium) on codon occupancy. We further considered whether *pmt1*^+^ overexpression caused methylation of other tRNA substrates. Only one weak methylation target was observed: An increase from 1.5 to 9.5 and 13.3% methylation of C38 in tRNA^Glu^_CUC_ and tRNA^Glu^_UUC_, respectively (Supplementary Table S5, Figure S2A), and there were no additional methylation sites in the presence of Q (Supplementary Figure S2B). In agreement with the moderate size in methylation changes, the respective codons (GAG and GAA) showed little change in codon occupancy upon *pmt1*^+^ overexpression (Figure 3B), which argues that the additional substrate of Dnmt2/ Pmt1 does not account for the translational effects on non-Q codons. Several of the tRNAs whose codons are affected by m^5^C38 (AGG (Arg), ACG (Thr) and AUA (Ile)) carry a threonyl-carbamoyl-adenosine (t^6^A) and cyclic t^6^A (43)) at position 37 next to the anticodon, suggesting that alterations in this modification may account for the differences in codon occupancy (see below). Also, several of the most strongly affected codons are decoded by low-abundance tRNAs (e.g. AGG, ACG, UUA, AUA and CCG have 1–2 chromosomal tRNA copies), suggesting that minor fluctuations in tRNA pools may cause changes in codon occupancy.

In summary, our data indicate that m^5^C38 on tRNA^Asp^ has a minimal effect on codon occupancy at the Asp codons, and that *pmt1*^+^ overexpression causes pleiotropic effects that may be related to cultivation conditions in minimal medium.

### Q modification suppresses translation misreading of the near-cognate GGC codon by tRNA^Asp^_QUC_

**Figure 4.**
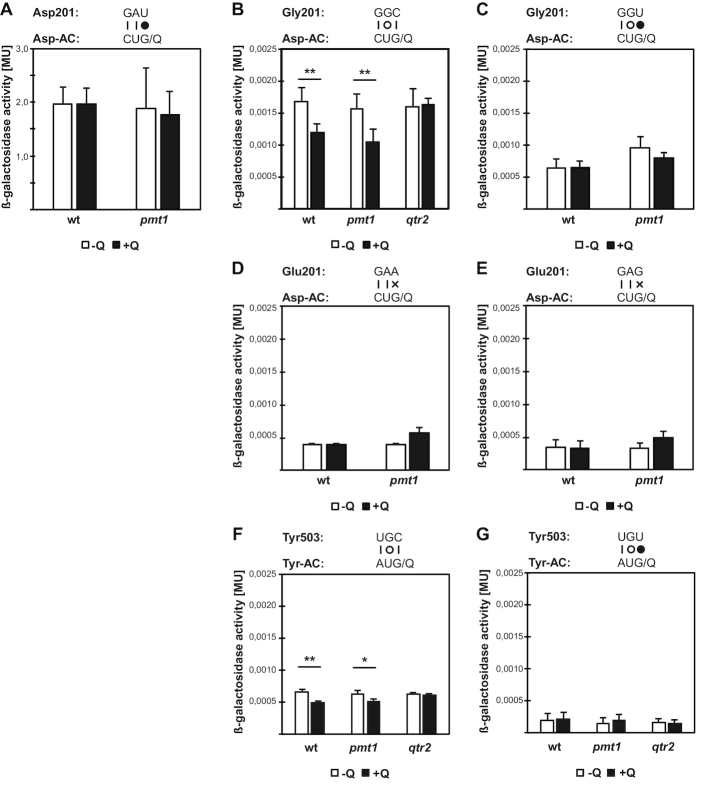
Misreading errors by tRNA^Asp^ of the Gly codon GGC, but not GGU, decrease in the presence of Q34 modification. The activity of wt *Escherichia coli* β-galactosidase expressed in *S. pombe* wt or *pmt1Δ* strains was measured in the absence (white bars) or presence of queuine (black bars, mean ± SD). The codon–anticodon pairing required for a (mis-)reading event is diagrammed above the graph. The upper line represents the codon, the lower line the anticodon (AC). Vertical lines represent Watson–Crick pairs, filled circles canonical wobble pairs, and open circles pairs requiring a tautomeric shift to form. (**A**) Activity of wt β-gal (wt, n = 5; *pmt1Δ, n* = 4). (**B**) β-gal activity of mutant version in which the Asp codon 201 is replaced by the Gly codon GGC (wt, *n* = 5; *pmt1Δ, n* = 6). (**C**) as in B, but Asp 201 replaced by the GGU Gly codon (*n* = 3). (**D, E**) Activity of β-gal with Asp 201 replaced by the Glu codon GAA or GAG. (**F, G**) Activity of β-gal with Tyr 503 replaced by the Cys codon UGC or UGU (*n* = 4). **P < 0.005; *P < 0.01. All other comparisons of –/+Q were statistically not significant.

We next asked how the loss of Q34 or m^5^C38 modifications affected the frequency of translation errors by adapting a β-galactosidase (β-gal) reporter system to measure misreading in *S. pombe* (16,44). Mutant versions of β-gal were used in which active sites are replaced by a different codon (and hence amino acid), which strongly reduces the activity of the enzyme. Rare events of codon misreading can result in the insertion of the wild-type amino acid at the mutant site, which restores enzymatic activity. Thus, measurement of β-gal activity from the mutant version allows the determination of the frequency of erroneous incorporations of the wild-type amino acid. We focused on erroneous reading by tRNA^Asp^, which carries both the Q34 and m^5^C38 modifications. Versions of β-gal were expressed in *S. pombe* in which the active-site Asp 201 codon (GAC/ GAU) is replaced by a near-cognate Gly (GGC/ GGU) codon, which requires formation of a U-G tautomer at the second position for misreading by tRNA^Asp^. As expected, a wt β-gal version gave a high level of enzymatic activity (Figure 4A), which was unaffected by the presence of Q or Dnmt2/ Pmt1, whereas the activity was much lower for the mutant β-gal versions. Significantly, the Gly codon mutant GGC showed lower enzymatic activity in the presence of Q than in its absence (Figure 4B), which suggested that Q modification may suppress erroneous incorporation of Asp by tRNA^Asp^ at this codon. Indeed, deletion of one of the subunits of the *S. pombe* eTGT complex (*qtr2Δ*) abrogated the Q-mediated error suppression, showing that Q incorporation into tRNA^Asp^ was required for suppression. Interestingly, the error suppression was not observed with the related Gly codon GGU (Figure 4C). Thus, Q-modification destabilizes second-position misreading by tRNA^Asp^_QUC_, but only in the context of the C-, not the U-ending glycine codon, suggesting that the G-C pairing at the wobble position contributes to mis-incorporation.

In contrast to the Gly GGC codon, the Glu substitutions GAA and GAG showed no differences in error rates (Figure 4D and E), indicating that Q modification is not essential to block wobble misreading by tRNA^Asp^_QUC_ of these codons.

We also tested misreading by tRNA^Tyr^_(GUA)_, another Q-modified tRNA, by mutating the Tyr 503 codon of the lacZ reporter. Interestingly, as for tRNA^Asp^, second-position misreading of the Cys codon UGC was suppressed in the presence of Q in wt and *pmt1Δ*, but not in *qtr2Δ*, whereas UGU was unaffected (Figure 4F and G), arguing that this is a general feature of Q modification in *S. pombe*.

### Genome-wide effects of Q and Dnmt2 on translation and mitochondrial function

**Figure 5.**
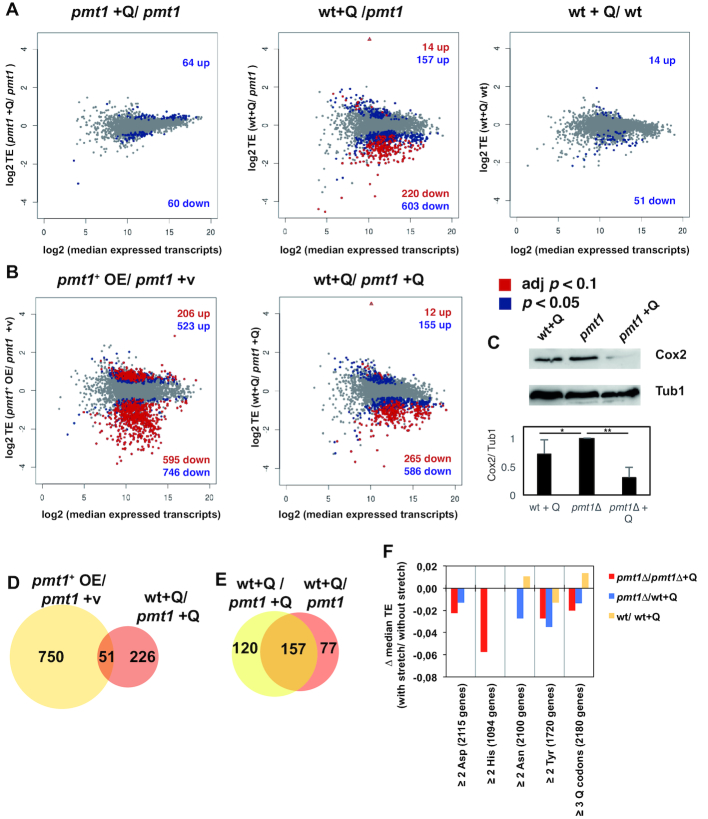
Effect of Q and m^5^C38 modification on translational efficiency. (**A**) Effects of Q on translational efficiency. Plots of the log_2_-fold change in translational efficiency of two compared conditions are shown relative to the log_2_ of the median expression of transcripts from the two conditions. Left, TE of *pmt1*Δ +Q/*pmt1*Δ; middle, TE of wt +Q/ *pmt1*Δ; right, TE of wt +Q/wt. Colours indicate transcripts with significantly different translational efficiency. Blue dots, *P* < 0.05, red dots, Benjamini–Hochberg adjusted (adj) *P* < 0.1. The number of genes in the respective *P*-value categories that are up- or down-rgeulated are given in the upper right and lower right corner of the plots, respectively. The triangle represents a data point that is outside the plotted frame and corresponds to *pmt1*^+^. (**B**) Effect of C38 methylation by Dnmt2/ Pmt1 on translational efficiency. The log_2_-fold change in TE of *pmt1*^+^ overexpression (OE)/*pmt1*Δ + vector (v) (left) and wt +Q/ *pmt1*Δ+Q (right) is plotted against the log_2_ median expression of transcripts of the two conditions. Representation as in (A). (**C**) Levels of Cox2 protein were decreased in *pmt1*Δ + Q. Protein extracts were separated by SDS-PAGE and analysed by Western blotting using an antibody against Cox2 (top) and, as a control, with an α-Tubulin antibody (Tub1). Below, quantification of Cox2 levels relative to Tub1 (mean ± SEM, *n* = 3, normalized to *pmt1*Δ value. *P = 0.11; **P = 0.002). (**D**) Overlap of differentially translated mRNAs between *pmt1*^+^ OE/ *pmt1*Δ +v (see Figure 4B, left) and wt +Q/ *pmt1*Δ +Q (Figure 4B, right) (adj. *P* < 0.1). (**E**) Overlap of mRNAs differentially translated in wt +Q/ *pmt1*Δ (Figure 4A, middle) and wt +Q/ *pmt1*Δ +Q (Figure 4B, right). (**F**) Translational efficiency of genes with stretches of Q codons was decreased in the absence of Q. The translational efficiency (TE) of genes with at least one stretch of 2 consecutive Asp, His, Asn or Tyr codons or three or more consecutive Q codons (size of the group of genes is given in parenthesis) was compared to that of all other genes not fulfilling this condition. The difference between the median translational efficiency of genes with the respective stretch compared to that of genes without the stretch is given. Only values that are statistically significantly different (Benjamini–Hochberg adjusted *P* < 0.05) are shown.

We next asked how Q and Dnmt2 modification of tRNAs affected the translation of individual mRNAs. To do so, the differential translational efficiency (TE) was determined by calculating the ratio of ribosome footprints relative to mRNA abundance (as measured by high-throughput RNA sequencing) (37) (Figure 5A and B). We first considered the effect of Q on translation by investigating differences in TE in the following comparisons: *pmt1*Δ+Q/ *pmt1*Δ (where only Q changes, no m^5^C38), wt + Q/*pmt1*Δ (both Q and m^5^C38 change) and wt + Q/wt (Q changes from 100% to 0%, m^5^C38 changes from 100% to 15%, Figure 5A). Of note, there were no statistically significant changes in levels of single mRNAs in these comparisons (adjusted *P*-values < 0.1), indicating that Q did not affect transcription.

The analysis of TE revealed that, upon addition of Q, wt+Q/*pmt1*Δ showed the most changes in TE, which is consistent with the fact that both modifications change (Benjamini–Hochberg-adjusted *P*-value < 0.1, Figure 5A, red dots). In fact, in *pmt1*Δ +Q/ *pmt1*Δ and wt+Q/wt, only few genes were differentially translated (*P* < 0.05, Figure 5A, blue dots), and none passed a more stringent statistical threshold (Benjamini–Hochberg-adjusted *P*-value < 0.1, Figure 5A). Also, the majority of mRNAs in wt+Q/ *pmt1*Δ showed a decrease in TE (Figure 5A, see numbers in plots; Supplementary Tables S6–S8). Investigation of the enrichment of gene ontology (GO) terms among the respective genes in wt +Q/*pmt1*Δ (adj. *P* < 0.1) using DAVID (45) revealed several GO categories related to ribosomal function, such as ribosomal protein genes and genes involved in rRNA processing as being enriched among the differentially translated mRNAs (Supplementary Figure S3A), and these genes were down-regulated in wt+Q relative to *pmt1*Δ (Supplementary Table S7). GO term analysis for *pmt1*Δ+Q/ *pmt1*Δ using the genes with differential TE (unadjusted *P* < 0.05) revealed enrichment for the same categories (Supplementary Figure S3A). In conclusion, our data indicates a mild down-regulation of the translation of selected ribosomal protein genes in the presence of Q34 and m^5^C38.

To obtain further information about functional groups of genes within the differentially translated mRNAs, the significantly mistranslated mRNAs from the wt+Q/*pmt1*Δ comparison (adj. *P* < 0.1) were subjected to Ingenuity Pathway Analysis (IPA, www.ingenuity.com), in which the human orthologs of the respective *S. pombe* genes are investigated for GO term enrichment. In contrast to DAVID (45), IPA utilizes not just a list of genes, but also takes into account the fold-change and *P*-value and thus has the potential to reveal associations that are missed by DAVID. IPA analysis showed a significant enrichment of categories related to ribosomal function, e.g. eIF2 signalling, eIF4 and p70S6K signalling as well as tRNA charging, as was observed using DAVID (Supplementary Figure S3B). It additionally revealed categories related to mitochondrial function, namely oxidative phosphorylation and mitochondrial dysfunction, which were undetected in DAVID. For instance, translational efficiency of the genes predicted to encode cytochrome c oxidase 1 (*cox1*), cytochrome b (*cob1*) and cytochrome *c* oxidase 2 (*cox2*) was strongly reduced in wt+Q compared to *pmt1*Δ (Table 1), suggesting misregulation of mitochondrial function in the presence of Q34 and m^5^C38 modification. Of note, these ribosome footprints were detected in our ribosome profiling experiments despite the fact that we used a protocol that did not enrich for mitochondrial ribosomes (46).

**Table 1.** Reduced translational efficiency of genes with a mitochondrial function in the presence of Q

Common name	log_2_FC (wt+Q /*pmt1*Δ), adj. *P* < 0.1*	log_2_FC (*pmt1*Δ+ Q/*pmt1*Δ), *P* < 0.05	log_2_FC (wt+Q/wt), *P* < 0.05	log_2_FC (*pmt1*^+^ OE/*pmt1*Δ), adj. *P* < 0.1	log_2_FC (wt+Q/ *pmt1*Δ+Q), adj. *P* < 0.1	Function
cox1	*−*3.85	-**	-	-	-	Cytochrome *c* oxidase 1 (predicted)
atp7	*−*0.98	-	-	-	-	F0-ATPase subunit D (predicted)
qcr6	*−*1.89	-	*−*1.09	*−*1.04	-	Ubiquinol–cytochrome *c* reductase complex subunit 8, hinge protein (predicted)
cob1	*−*4.44	*−*1.85	-	-	-	Cytochrome *b*, Cob1 (predicted)
cox8	*−*1.60	-	-	-	-	Cytochrome *c* oxidase subunit VIII (predicted)
cox2	*−*4.58	*−*3.024	-	-	-	Cytochrome *c* oxidase 2 (predicted)

*log_2_-fold change (log2FC) in translational efficiency of comparisons (e.g. wt+Q/*pmt1*Δ) is given. The significance level is indicated (Benjamini–Hochberg-adjusted (adj.) *P*-value < 0.1 or unadjusted *P* < 0.05).

**(−) indicates no significant change in TE in the two conditions with the indicated *P*-value.

To validate the reduction in translation of genes with a mitochondrial function using an orthogonal method, we determined Cox2 protein levels by Western blotting (40). In agreement with the results from ribosome profiling, reduced levels of Cox2 were observed in cells grown in the presence of Q, especially in *pmt1Δ* (Figure 5C). While this decrease was not statistically significant in wt + Q by western blotting (Figure 5C, bottom), evaluation of the levels of newly synthesized mitochondrial proteins by *in vivo* labeling with ^35^S-marked amino acids (47) showed reduced levels of Cox2 and Cob1 in both wt and *pmt1*Δ cells treated with Q (Supplementary Figure S3C). Altogether, these results supported the notion that the presence of Q-modified tRNAs resulted in a mild reduction in translation of a subset of mRNAs with a mitochondrial function.

We next investigated how m^5^C38 modification by Dnmt2/ Pmt1 affected translation of mRNAs by evaluating the difference of TE in the comparisons *pmt1*^+^ OE/ *pmt1*Δ + v (i.e. the difference between 100% and no m^5^C38 in the absence of Q, Supplementary Table S9) and wt + Q/ *pmt1*Δ + Q (i.e. the difference between 100% and no m^5^C38 in the presence of Q, Supplementary Table S10). On the transcriptional level, *pmt1*^+^ overexpression caused the upregulation of 43 genes (including *pmt1*^+^ itself, Benjamini–Hochberg adjusted *P*-value <0.05, Supplementary Figure S3D), and no genes were significantly down-regulated. This indicated that *pmt1*^+^ overexpression mostly acted on translation rather than transcription.

Of the two comparisons, the more prominent differences in TE were observed upon *pmt1*^+^ overexpression, where more mRNAs showed reduced TE in the presence of m^5^C38 (Figure 4B). The effects of m^5^C38 by *pmt1*^+^ overexpression were more pronounced than those of changing m^5^C38 methylation in the presence of Q (wt+Q/*pmt1*Δ+Q) (Figure 5B), which may be due to the effect of overexpression as well as to the absence of Q in the *pmt1*^+^ OE/ *pmt1*Δ+v comparison. Notably, *pmt1*^+^ overexpression is also the condition where the strongest pleiotropic effects on codon occupancy were observed (Figure 3). In total, 51 genes showed a shared change in TE between *pmt1*^+^ OE/ *pmt1*Δ +v and wt+Q/ *pmt1*Δ (Figure 5D, 48 down-regulated, 3 upregulated), among which genes with transmembrane transporter activity were enriched (e.g. ammonium transmembrane transporter gene *amt1*^+^ and the hexose transmembrane transporter gene *ght1*^+^, Supplementary Tables S7 and S9), the functional role of which remains to be seen.

Closer inspection of the differentially translated mRNAs also revealed that two genes related to t^6^N and ct^6^A modification show changes upon *pmt1*^+^ overexpression (Supplementary Table S9). Specifically, the *S. pombe* homolog of Sua5, which is required for t^6^A synthesis (48), is less abundant at high Dnmt2/ Pmt1 levels (log2-fold change (*pmt1*^+^ OE/*pmt1Δ*) = −0.971), and Tcd1, the tRNA threonyl–carbamoyl–adenosine dehydratase required for ct^6^A production (43) is more highly translated (log2-fold change (*pmt1*^+^ OE/ *pmt1Δ*) = 1.096). One can therefore speculate that t^6^N modification of tRNAs is reduced (based on lower levels of Sua5) relative to ct^6^A modification, which may be enhanced due to higher Tcd1 levels, thus causing an imbalance in t^6^N and ct^6^A modification of the respective tRNAs. This is particularly relevant with respect to the observation that the codons of several t^6^A/ ct^6^A-modified tRNAs (i.e. ANN codons, e.g. AGG (Arg), ACG (Thr) and AUA (Ile)) display lower codon occupancy upon *pmt1*^+^ overexpression (Figure 3), lending support to the notion that subtle changes in the balance of these modifications elicit the changes in codon occupancy.

We also considered the mRNAs that were differentially translated both in wt +Q/ *pmt1*Δ +Q (Figure 5B, right plot) and wt+Q/*pmt1*Δ (Figure 5A, middle plot), which gives an indication as to which genes are affected by m^5^C38 (in the presence of Q) and which by both m^5^C38 and Q. There was an overlap of 157 genes (Figure 5E), among which genes with ribosomal function were significantly enriched (38 genes, *P* < 0.05), indicating that m^5^C38 alone reduced the translation of a group of ribosomal protein genes. However, the translation of mitochondrial targets was only down-regulated in the wt+Q/ *pmt1*Δ, but not the wt+Q/ *pmt1*Δ +Q dataset (Table 1, Supplementary Tables S7 and S10), showing that the regulation of mitochondrial function was specific to Q, but not m^5^C38 modification.

We further asked what distinguishes the set of differentially translated mRNAs from non-affected mRNAs. There was no correlation with content of Asp, His, Asn or Tyr codons, but genes with at least one stretch of two or more consecutive Asp, His, Asn or Tyr codons or three or more of any of these codons showed a minimal, but statistically significant trend towards decreased TE in the absence of Q as compared to genes without the stretches (Benjamini–Hochberg-adjusted *P* < 0.05, Supplementary Figure S5F). The exception to this was the wt/wt+Q comparison, where TE was increased on genes with Asn or Q codon stretches, but this comparison is complicated by the fact that, next to the change of Q modification, m^5^C38 also changes from 15 to 100%. In summary, the net effect of Q modification across the transcriptome was very moderate and showed at most a mild improvement of the efficiency of translation on mRNAs with stretches of Q codons in the presence of Q.

### Q34 modification impaired mitochondrial function in *S. pombe*

**Figure 6.**
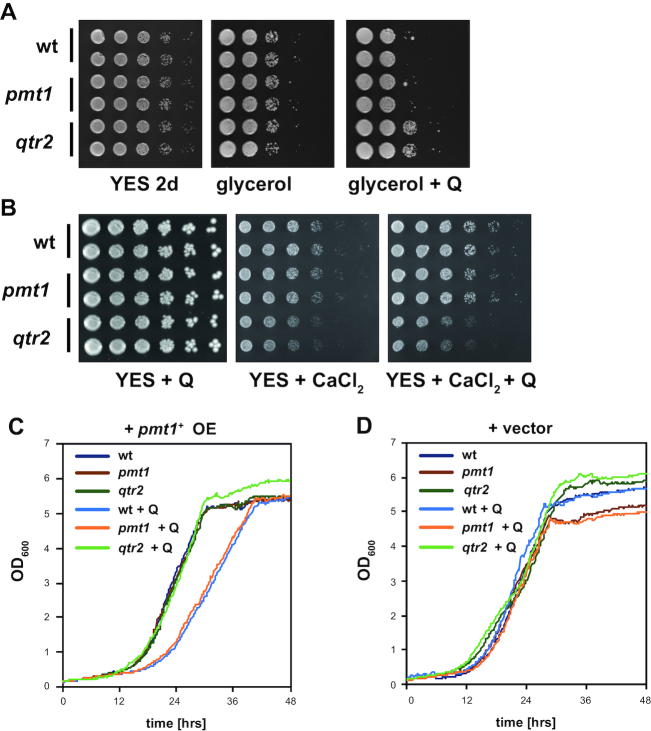
Q modification caused a respiratory defect in *S. pombe* cells and a growth defect when *pmt1^+^* was overexpressed. (**A**) Serial dilutions of wt, *pmt1Δ* and *qtr2Δ S. pombe* strains were spotted on full medium (YES) with glucose and on medium with 3% glycerol in the presence or absence of Q. (**B**) Strains as in (A) were grown on medium with 300 mM CaCl_2_ with our without Q. (**C**) Overexpression of *pmt1^+^* caused a growth defect in the presence of Q in wt and *pmt1Δ*, but not *qtr2Δ*. (**D**) No growth defect of the strains was observed with the vector control.

The differential effects of Q and Dnmt2 tRNA modification on translation raised the question whether the translational differences caused phenotypic differences under different growth conditions (Supplementary Table S11). To test an effect on mitochondrial function, as was suggested from the above results of Q and m^5^C38 modification on TE of genes with a mitochondrial function, *S. pombe* cells were grown with glycerol instead of glucose as a carbon source, since defects in mitochondrial respiration prevent growth on glycerol (49). Interestingly, the cells showed a slow growth phenotype on glycerol medium that was exacerbated by the presence of Q, suggesting impaired mitochondrial function in the presence of Q (Figure 6A). This was in agreement with the observation that Q modification reduced translation of proteins with a mitochondrial function (Table 1). Furthermore, the growth defect on glycerol medium was not observed in *qtr2Δ* cells, which lack *S. pombe* eTGT, showing that Q incorporation into tRNAs disturbed mitochondrial function (Figure 6A). Also, *S. pombe* cells showed slow growth on medium containing CaCl_2_, an indicator for disturbed mitochondrial function in *S. cerevisiae* (50), and this was suppressed by supplementing the medium with Q in wt and *pmt1Δ*, but not in *qtr2Δ* cells (Figure 6B). We furthermore observed a moderate but significant reduction in mitochondrial membrane potential in the presence of Q in *pmt1Δ* cells, but, for unknown reasons, not in wt + Q (Supplementary Figure S3E), even though wt and *pmt1*Δ showed similar defects in growth on glycerol +Q. Taken together, these results suggested that mitochondrial function was mildly impaired by Q modification of tRNAs.

Furthermore, we observed that upon overexpression of *pmt1^+^*, the addition of Q caused a decreased growth rate of *S. pombe* cells. Again, this growth-inhibitory effect of Q depended on its incorporation into tRNAs, because the effect was abrogated in *qtr2Δ* cells (Figure 6C and D). Interestingly, this defect was not seen when *pmt1*^+^ was expressed from the chromosomal gene. Possibly, Dnmt2/ Pmt1 at high levels binds and sequesters non-target tRNAs, or leads to the modification of RNAs other than tRNAs whose methylation causes the growth defect.

### *In vivo* stimulation of Dnmt2-dependent tRNA^Asp^ methylation by queuosine incorporation is conserved in mice

Our previous work showed that Dnmt2-dependent tRNA methylation in *S. pombe* is stimulated by prior incorporation of Q into tRNA^Asp^ by the eTGT enzyme (17), raising the question whether it was an unusual property of the yeast Dnmt2/ Pmt1 enzyme, or whether the Q dependence extends to other homologs. The yeast enzyme carries a non-consensus serine residue in the catalytic motif IV (sequence PSCQ in *S. pombe*, PPCQ in other homologs), which earlier had been proposed to inactivate the enzyme (51).

**Figure 7.**
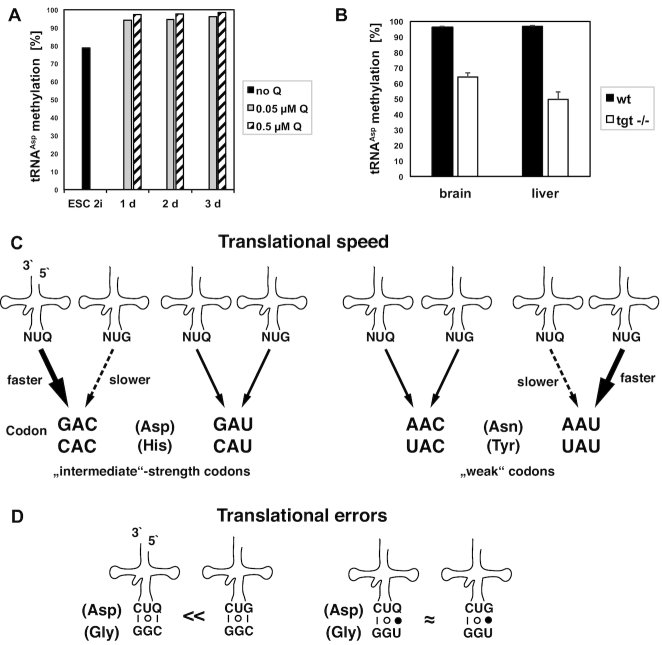
Stimulation of Dnmt2-dependent m^5^C38 methylation by Q is conserved in mice. (**A**) Mouse embryonic stem cells were cultivated in 2i medium and treated for 1, 2 or 3 days with 0.05 or 0.5 μM Q. RNA was extracted, and m^5^C38 levels in RNA^Asp^ were determined by high-throughput bisulfite sequencing. (**B**) m^5^C38 levels in RNA^Asp^ were determined as in (A) in RNA extracted from brain or liver tissues from wt or *Qtrd1−/−* mice (*tgt −/−*). Mean ± SD is reported (n = 3). (**C**) Graphical summary of the effect of tRNA Q modification on translational speed in *S. pombe*. The top row shows the tRNAs carrying either Q34 or G34. They translate the respective C- or U-ending codons. Q modification results in faster translation of the C-ending codons for Asp and His (bold arrow; ‘intermediate-strength’ codons according to (4)), but has no effect on the respective U-ending codons. Q modification slows down translational speed of the U-ending codons for Asn and Tyr (dashed arrow, ‘weak’ codons according to (4)). (**D**) Graphical summary of the effect of Q modification on suppressing translational errors. Q modification of tRNA^Asp^ reduces misreading of the C-ending, but not the U-ending Gly codon.

Here, we sought to test whether m^5^C38 methylation in mouse cells depended on Q supplementation or Q incorporation by eTGT. For this purpose, mouse embryonic cells (mESCs) were cultivated in the serum-free, feeder-free 2i medium (52), which is devoid of external queuine or queuosine sources. Importantly, the methylation level was increased from 78.9% in the absence of queuine to ca. 96% and 98.3% in its presence (with 0.05 or 0.5 μM Q) (Figure 7A), indicating that the stimulation of Dnmt2 by Q is conserved in mice. We also investigated m^5^C38 methylation of tRNA^Asp^ in mouse tissues lacking the eTGT complex, which exchanges G34 for Q34 in tRNAs. Such mice lack Q-modified tRNAs (23). In brain and liver tissues of wild-type mice, the methylation levels were approx. 95%. This level was decreased to 62% and 45% in the two tissues from mice homozygous for a gene trap insertion in *Qtrt1* (Figure 7B), which encodes a subunit of eTGT (23). This showed that m^5^C38 methylation in tRNA^Asp^ strongly depended in Q incorporation into the tRNA by eTGT in mouse tissues.

We furthermore investigated how mutation of the non-consensus serine of Dnmt2/ Pmt1 in *S. pombe* to a consensus proline (*pmt1-S80P*) affected activity and Q-dependent stimulation of the enzyme *in vivo. pmt1-S80P* cells showed 20% m^5^C38 in tRNA^Asp^ in the absence, and this level was increased to 42% when cells were cultured with Q (Supplementary Figure S5). Thus, the mutant Dnmt2/ Pmt1 retained activity, but was less efficiently stimulated by Q than the wild-type enzyme.

## DISCUSSION

The discovery of queuosinylation at the wobble position of tRNAs dates back to 1972 (7), yet the physiological role of this modification in translation has been unclear (10,12). Here, we present ribosome profiling data to address how Q modification, in conjunction with Dnmt2-dependent m^5^C38 methylation, modulates translation across the genome of *S. pombe*.

Our analysis allows several important conclusions. First, we observed that Q modification increases the translational efficiency of C-ending Q codons relative to U-ending codons in *S. pombe*. This finding was unexpected in light of the fact that Q modification has long been postulated to compensate for the weaker codon–anticodon interaction of Q–U base pairing relative to G-C pairing (14). The present results are, however, in agreement with a study of codon usage across *Drosophila* species that reported a preference for C- over U-ending codons at evolutionarily conserved Q codon sites (15). Our data show that Q affects the translational speed transcriptome-wide, by accelerating C-ending codons of His and Asp, and by decreasing the speed of the U-ending codons for Asn and Tyr (Figure 7C). In this manner, the Q modification contributes to the harmonization of translational speed across different codons of the genetic code.

Second, our data fit well with the theory that codon–anticodon strength is equilibrated across the genetic code (4). We find that Q modification differentially affects translation of codons that are considered ‘weak’ (e.g. AAU and UAU, Asn and Tyr) relative to ‘intermediate’ codons (e.g. CAC, GAC, His and Asp), based on their base-pairing properties at the first two positions of the codon. According to this view, the ‘stronger’ codons, which at the first two positions have a G-C pair (CAC and GAC), will prefer the U-ending codon, and the translation of the C-ending codon is affected by Q34 modification of the tRNA. Conversely, the ‘weaker’ codons, which have only A-U at the first two bases of the codon (AAU, UAU), will prefer the C-ending codon, and the U-ending codon is affected by Q-modification of the tRNA.

How do translational effects of Q in *S. pombe* relate to those in higher eukaryotes? Importantly, our recent work shows that Q modification in human cells fundamentally serves the same purpose, namely to adjust translational speed of C- and U-ending Q codons (13). However, contrary to *S. pombe*, the absence of Q in human cells causes a reduction of translational speed at most Q codons, and Q modification increases translational speed of the U- relative to the C-ending codons, whereas it increases the speed of the C-ending codon in *S. pombe*. This is particularly interesting in light of the fact that the relative usage of C- to U-ending codons differs between the human and *S. pombe* genomes (human: C:U ca. 55%: 45%; *S. pombe*: C:U ca. 38%: 62%) (53). In either case, the net result of Q modification is to equilibrate translational speeds between C- and U-ending Q codons. Thus, the global effect shows a striking convergence in the two evolutionarily distant systems, which at the same time implies species-specific differences in the mechanism employed to achieve this. One mechanism may be the further modification of Q to galactosyl- and mannosyl-Q in mammalian cells (54). Another possibility is that the tRNAs of the two organisms differ in the ‘proximal extended anticodon’ (4), especially positions 37 and 38 at the 3′ side of the anticodon, and positions 31/ 39, which form the base of the anticodon stem. The identity and modifications of these positions is critical for base stacking and stabilization of codon–anticodon pairs. Notably, *S. pombe* tRNA^Tyr^ carries A37, which is hypermodified (i^6^A37/ t^6^A37) to improve stacking with A36 of the anticodon (55). However, human tRNA^Tyr^ carries 1-methyl-G37 (m^1^G), which has less favourable stacking with A36. Such differences in stacking may thus explain why Q-modification shows a differential effect on Tyr codons in human cells and fission yeast.

Interestingly, Q modification differs in its effect on codon occupancy from other tRNA modifications. A recent study investigated codon occupancy in mutants of *S. cerevisiae* lacking tRNA-modifying enzymes and found that the majority of mutants (with some notable exceptions) show increased ribosomal occupancy at the corresponding codons and thus slowing of translation on those codons (41), which mirrors earlier observations of the absence of Elongator in *S. cerevisiae* and *C. elegans* (56). Thus, Q modification so far is unique among tRNA modifications in that it causes differential effects on codons that differ in their wobble position.

Our study furthermore reveals that the Q modification suppresses misreading of the C-ending glycine and cysteine codons, but not the respective U-ending codons (GGC and UGC, but not GGU and UGU; Figure 7D). This again is in agreement with the hypothesis generated from the investigation of Q codon usage in Drosophilids that C-ending codons are evolutionarily advantageous, because they are less error-prone than the respective U-ending codons under conditions of high Q modification (15).

All the second-codon misreading pairings mentioned above require the generation of a tautomeric base at the second position (57). Our data indicate that this process is disfavoured if there is a Q-C pairing at the wobble position, whereas the tautomeric form is equally likely for G-U and Q-U pairing in the GGU codon. Possibly, Q34 leads to a distortion of the codon–anticodon mini-helix and thus hinders formation of the G-U pairing at the second position. An earlier study showed that Q34 in tRNA^Tyr^ causes distortion of this mini-helix and leads to the displacement of C in a first-position C-A mismatch (58). Of note, we did not observe an effect of Q on the decoding of the Glu codons, which differ in the wobble position from the Asp codons and whose misreading by tRNA^Asp^ can be hypothesized to be suppressed by Q. Intriguingly, these suppression effects on erroneous decoding by Q mirror those in *E. coli* with respect to tRNA^Asp^, but not tRNA^Tyr^ (16), arguing for important species-specific differences in role of Q in translational accuracy. One possibility is that this is due to differences in the further modification of Q-modified tRNAs in the different systems (54).

Our investigation of the role of Q and m^5^C38 on translational efficiency revealed relatively moderate effects on a limited number of genes. Prominent effects were observed for Q modification in reducing translation of several mRNAs with a mitochondrial function. Consistent with this, *S. pombe* cells showed a respiratory defect in the presence of Q. It will be interesting to see how this relates to other tRNA modifications, whose loss is often associated with mitochondrial defects (59). An interesting possibility is that Q not only shows cross-talk with Dnmt2/ Pmt1-dependent C38 methylation, but also affects other tRNA modifications that modulate the translatome. We also observed effects on the translation of a group of ribosomal protein genes, the source of which requires further investigation. Contrary to our observations in human cells (13), Q depletion in *S. pombe* did not cause a defect in protein folding and activation of the unfolded protein response.

An important question has been whether the Q-dependence of Dnmt2 activity is a peculiarity of *S. pombe*, or whether it is more common in eukaryotes. In agreement with our recent work in HeLa cells and mice (13), we find that m^5^C38 methylation of tRNA^Asp^ in mice strongly depends Q incorporation by eTGT, indicating conservation of Q as a determinant for Dnmt2 activity across homologs. The modulation of translational speed and accuracy by the Q and m^5^C38 modifications is particularly intriguing in light of the fact that eukaryotes scavenge Q from bacterial sources and absorb it in the gut. This is a striking example of how the availability of a nutritional supplement fine-tunes the speed and fidelity of the translational apparatus in the host.

## DATA AVAILABILITY

Ribosome profiling and RNA-seq data are available in the NCBI GEO database, record GSE102376.

## Supplementary Material

Supplementary Data
