# Supplementary material for: Queuine links translational control in eukaryotes to a micronutrient from bacteria
Source: Nucleic Acids Res. 2019 Feb 1;47(7):3711–27. doi: 10.1093/nar/gkz063 (PMC6468285; doi:10.1093/nar/gkz063)
Supplement: Supplementary Data [file gkz063_supplemental_files.zip › gkz063/Suppl_Info_230119.pdf]

Supplementary information

Quality control of ribosome profiling data

For the evaluation of the ribosome profiling data, we investigated the data in metagene plots. The ribosome-protected peak was located 12 nucleotides upstream of the start codon and exhibited the expected trinucleotide periodicity (Supplementary Figure S4A). Also, metagene plots showed the expected distribution of ribosome footprints across the ORF (Supplementary Figure S4B).

In order to stabilize ribosomes on the mRNA, cells were treated with the translational inhibitor cycloheximide (CHX) as in the original ribosome profiling protocol and many other studies (1-4). Since the use of CHX in *S. pombe* has previously been reported to affect the translational efficiency (TE, ribosome footprints relative to mRNA level) of ribosomal protein genes upon nitrogen starvation (5), we investigated the TE of this group of genes. They showed a TE comparable to that of all other genes (Supplementary Figure S4C, D), indicating that the effects on translation observed here were not due to CHX treatment (6,7). Also, effects of CHX have been illustrated by a negative correlation between the mean relative A-site enrichment of a codon and the inverse of its tRNA adaption index (tAI) (7). However, we found correlations that were close to 0 (mildly positive values, Supplementary Table S12), indicating that CHX did not have the same effect on translation in our experiments as in previous studies using CHX (7). Finally, we also queried our ribosome profiling data for whether there were changes in ribosome density at a particular offset upstream or downstream of a given codon. A previous analysis has shown that usage of CHX causes patterns of ribosome density downstream of different codons that were not present in data from experiments without CHX (7). However, we did not observe such patterns in our data (Supplementary Figure S4E), arguing that the results of our ribosome profiling reflected genuine features of translation, rather than an effect by CHX treatment.

Supplementary Figures

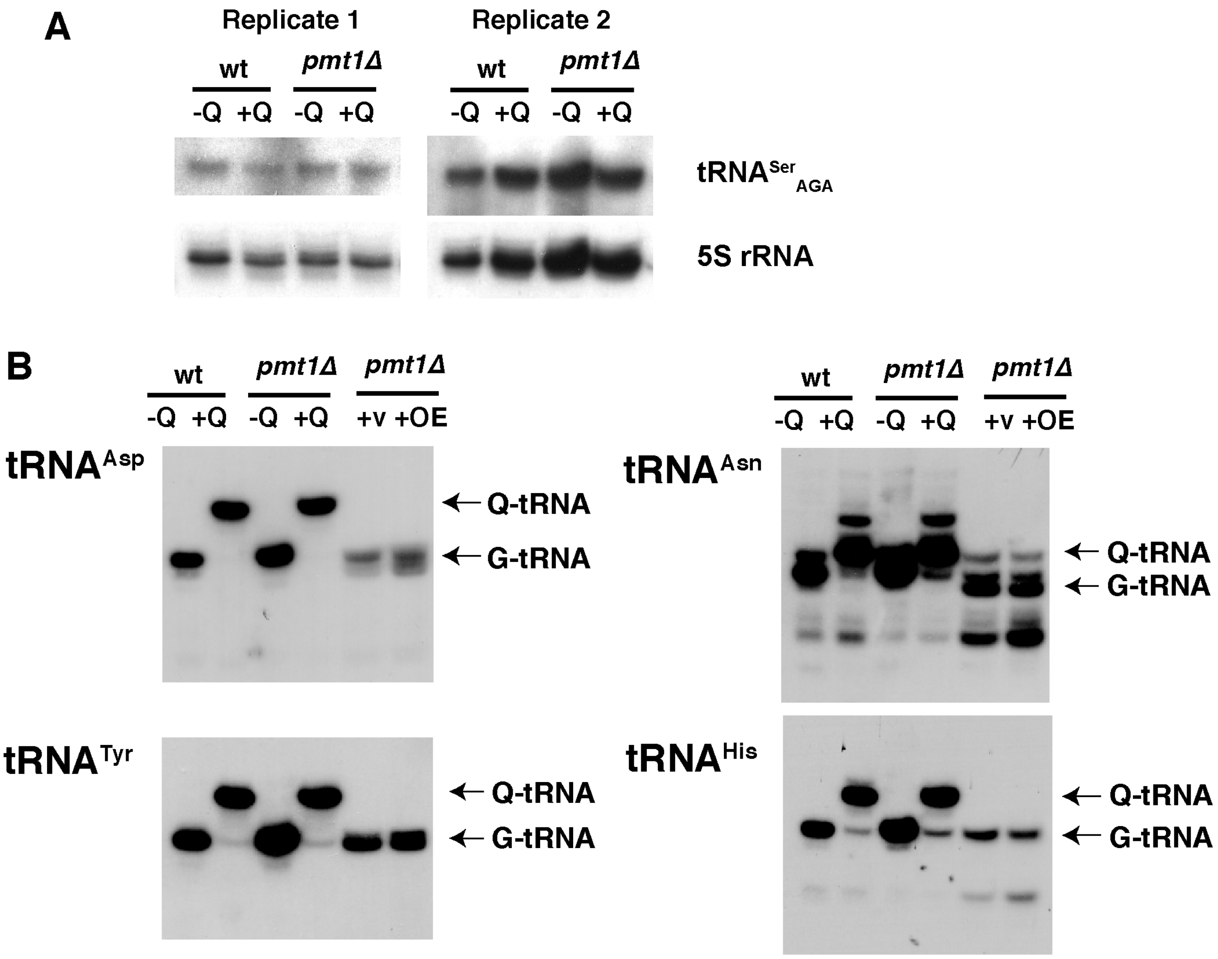

Supplementary Figure S1. Measurement of Q levels in tRNA using polyacrylamide gels covalently linked with N-acryloyl-3-aminophenylboronic acid (APB). (A) Measurement of $tRNA^{Ser}_{AGA}$ levels. Northern blotting with APB-gels was performed, and membranes were probed with a probe for $tRNA^{Ser}_{AGA}$ (top) and for the 5S rRNA (bottom, loading control). RNA samples were from wt and *pmt1*Δ with or without Q (two biological replicates). (B) Northern blotting with APB-gels was performed, and membranes were probed with a probe for the indicated tRNA probes. RNA samples from wt and *pmt1*Δ with or without Q and upon *pmt1*$^{+}$ overexpression (+OE; +v, vector control; both without Q) are shown.

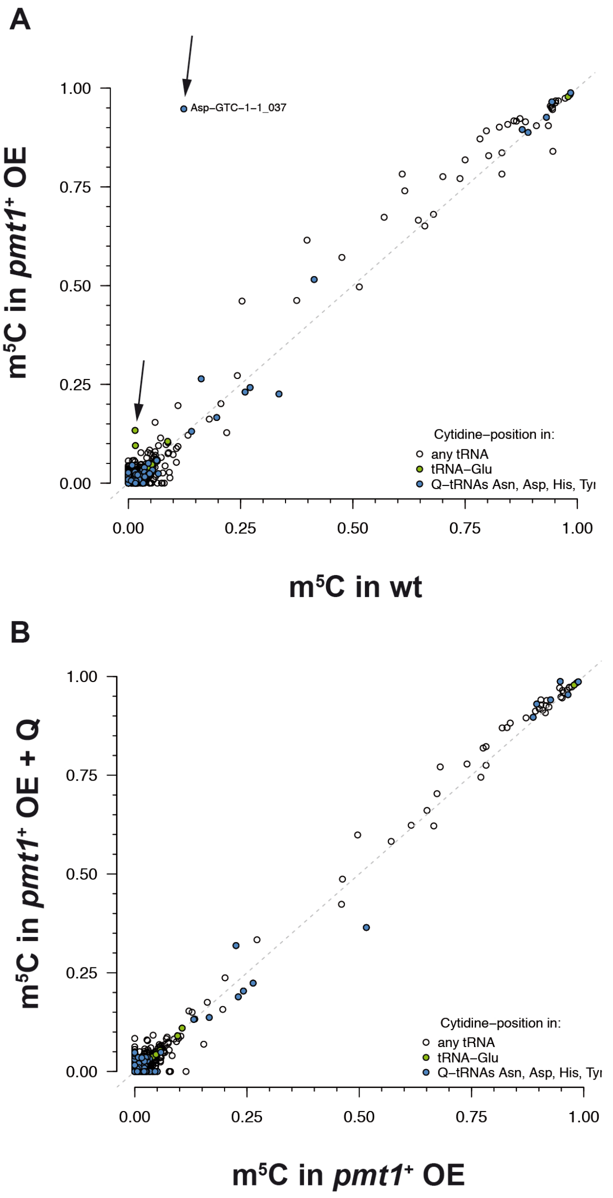

Supplementary Figure S2. *pmt1*$^{+}$ overexpression causes a mild increase in m^{5}C38 methylation of tRNA^{Glu}. (A) tRNA methylome analysis revealed tRNA^{Asp} and tRNA^{Glu} as Dnmt2/ Pmt1 targets upon *pmt1*$^{+}$ overexpression. Methylation levels as measured by RNA bisulfite sequencing of *S. pombe* wt cells grown in minimal medium (x-axis) were compared to those upon *pmt1*$^{+}$ overexpression (y-axis). *pmt1*$^{+}$ overexpression (OE) causes a significant increase of m^{5}C38 on tRNA^{Asp} and tRNA^{Glu} (arrows). (B) Comparison of tRNA methylome analysis of cells overexpressing *pmt1*$^{+}$ in the absence (x-axis) versus the presence (y-axis) of Q shows no significant changes in m^{5}C levels upon *pmt1*$^{+}$ overexpression in the presence of Q.

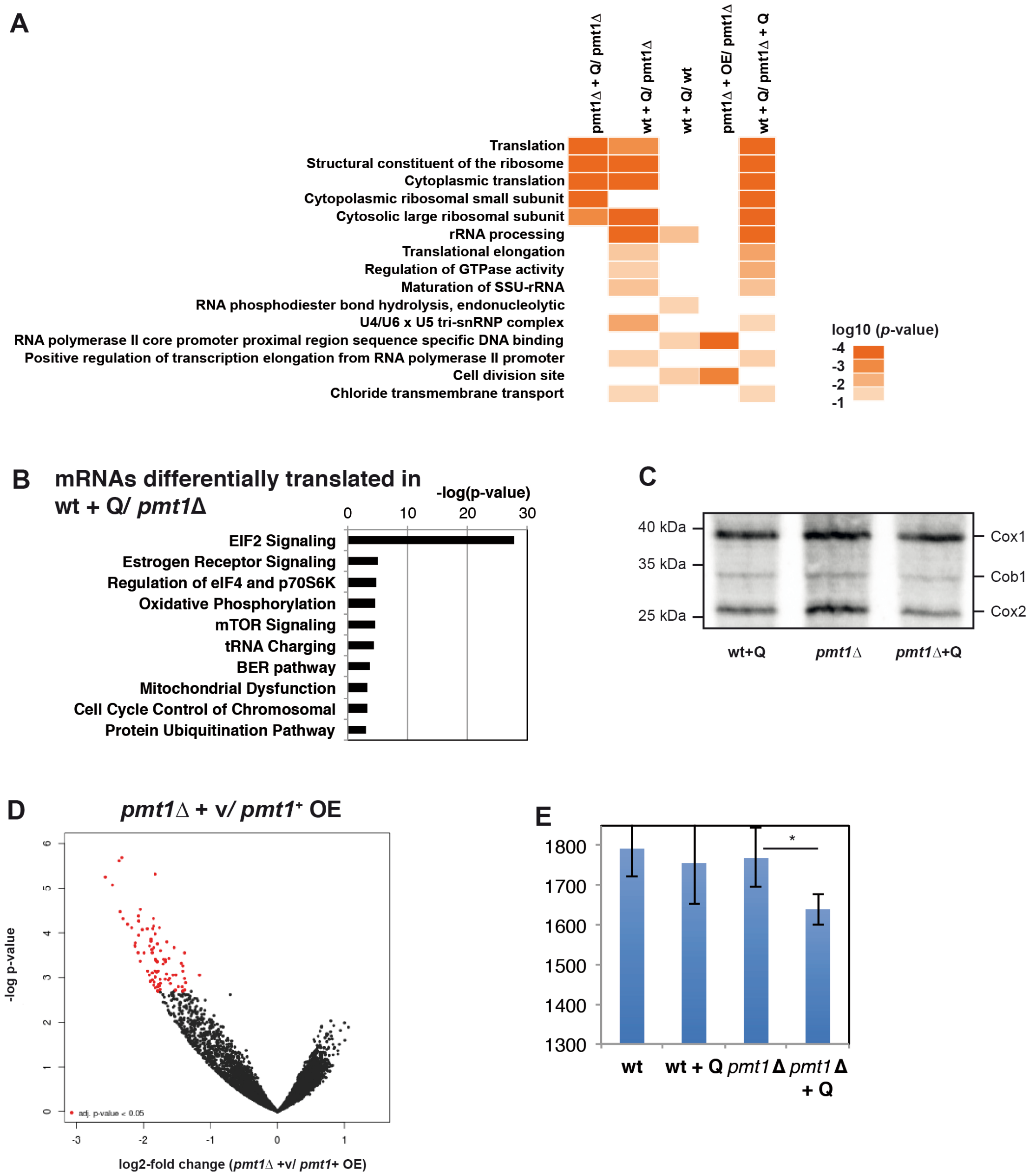

Supplementary Figure S3. Effects of Q and m^{5}C38 on translational efficiency. (A) GO term analysis of mRNAs differentially translated under the indicated conditions, as determined by DAVID analysis (8). *p*-values for the enrichment in the individual categories is indicated with the colour code on the right. (B) Ingenuity Pathway Analysis of human orthologs of *S. pombe* mRNAs differentially regulated in wt + Q/ *pmt1*Δ. p-values of the individual categories are indicated. (C) *In vivo* ^{35}S labelling of mitochondrial proteins reveals reductions of Cox2 and Cob1 in wt and *pmt1*Δ cells grown in the presence of Q. Amino acids were labelled with ^{35}S methionine/ cysteine in the presence of anisomycin, which blocks cytoplasmic translation, and equal amounts of protein was loaded on an SDS-PAGE gel. Proteins

were transferred to a nitrocellulose membrane, which was used to expose a Phosphorimager screen. (D) Differential mRNA expression levels upon *pmt1*$^{+}$ overexpression (OE) compared to *pmt1*Δ transformed with the control vector (+v). Log2-fold change in mRNA level (*pmt1*Δ+v/ *pmt1*$^{+}$ OE) is shown relative to the *p*-value. Genes with a Benjamini-Hochberg adjusted *p*-value < 0.1 are shown in red. They are expressed higher upon *pmt1*$^{+}$ OE. The *pmt1*$^{+}$ gene itself was excluded from the plot in order to better visualize the changes of other genes. (E) Mitochondrial transmembrane potential of *S. pombe* cells with the indicated genotypes cultured in YES with or without Q was measured using TMRE staining. Mean values ± SD (n=3) are given. *, *p* = 0.057.

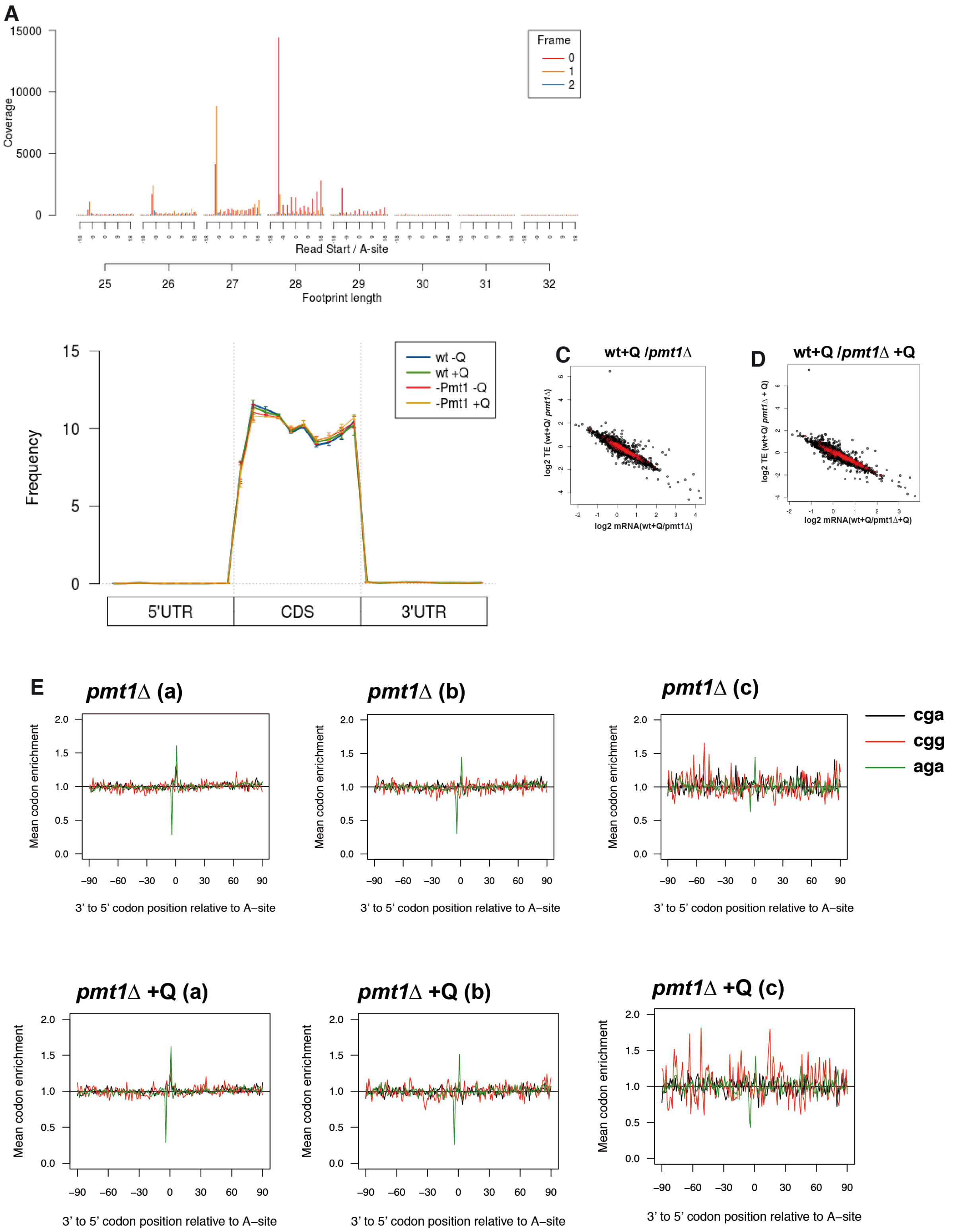

Supplementary Figure S4. Validation of ribosome profiling data. (A) Representative metaplot of ribosome footprints from wt *S. pombe* cells (AEP1, replicate a). 25-32 mer ribosome footprint reads were summed across all start codons. In-frame 27-30 mer ribosome footprints showed the correct periodicity. Of note, due to mRNA digestion, some reads start at frame 1 with an offset of 1 nt, for

example at length 27. This is accounted for in the subsequent codon assignment. (B) Representative metagene plot of 27-30 mer ribosome footprints of wt (+/-Q) and *pmt1*Δ (+/- Q). Values show mean ± SD (n=3). (C) Ribosomal protein genes showed translational efficiency comparable to that of all other genes. The log2-fold change in translational efficiency (TE) of wt+Q/ *pmt1*Δ is shown relative to the log2-fold change in mRNA levels of wt+Q/ *pmt1*Δ. Ribosomal protein genes are coloured in red. (D) as in (C), but for wt+Q/ *pmt1*Δ+Q. (E) Profiles of mean relative enrichments at a range of offsets around the arginine codons CGA (black), CGG (red) and AGA (green) in ribosome profiling data from three replicates of *pmt1*Δ (top, replicates a, b and c) and of *pmt1*Δ + Q (bottom, replicates a, b, c).

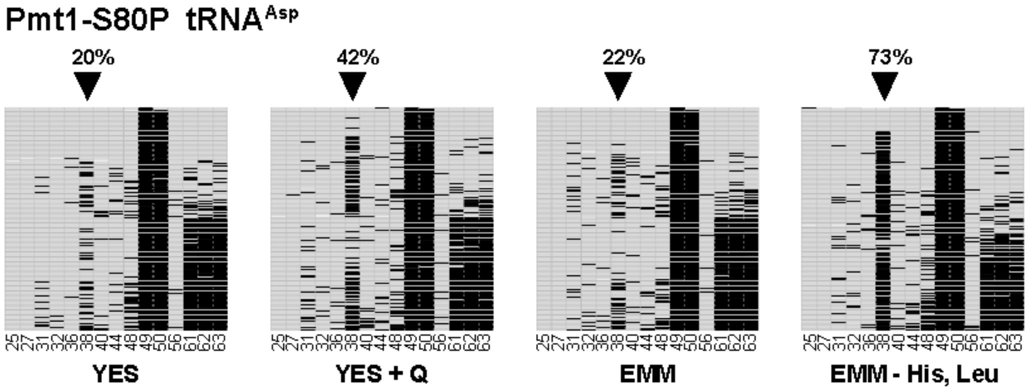

Supplementary Figure S5. A variant of Dnmt2/ Pmt1 in *S. pombe* with the non-consensus serine 80 mutated to a consensus serine (Pmt1-S80P) retains m^{5}C38 methylation activity and is stimulated by Q. High-throughput bisulfite sequencing of tRNA^{Asp} from a *pmt1*-S80P strain grown in full medium (YES) with or without queuine and in EMM minimal medium with or without histidine and leucine (amino acid starvation stimulates *pmt1* activity) as indicated was performed as described . Results from 1024 independent sequences are shown. Grey, unmethylated cytosine; black, methylated cytosine; white, mismatch. Arrows indicate the C38 position. Methylation levels are given in %.

Supplementary Table S1: *S. pombe* strains used in this study

Designation	Genotype	Source
AEP1	h⁻ *leu1-32 ura4-D18 his3-D3*	YGRC
AEP8	= AEP1; *pmt1Δ::kanMX*	(9)
AEP288	= AEP1; *qtr2*(SPAC2F3.13c)Δ*::NatMX*	(10)
AEP99	= AEP8 + REP4X	(9)
AEP100	= AEP8 + pAE1463	(9)
AEY490	h⁻ *leu1-32 ura4-D18 his3-D3 pmt1-S80P*	This study

Supplementary Table S2: Plasmids used in this study

Designation	Description	Source
pAE1428:	REP3X (*LEU2*, *nmt1* promoter)	ATCC/ S. Forsburg
pAE1429:	REP4X (*ura4*$^{+}$, *nmt1* promoter)	ATCC/ S. Forsburg
pAE1463:	REP4X-*pmt1*$^{+}$	(9)
pAE1592	REP3X-*pmt1*$^{+}$	(9)
pAE1437	pSLF173 (*ura4*$^{+}$, *nmt1* promoter)	ATCC/ S. Forsburg
pAE2562	pSLF173-LacZ-wt	This study
pAE2563	pSLF173-LacZ-D201-GAA	This study
pAE2564	pSLF173-LacZ-D201-GAG	This study
pAE2565	pSLF173-LacZ-D201-GGU	This study
pAE2566	pSLF173-LacZ-D201-GGC	This study
pAE2566	pSLF173-LacZ-D201-GGC	This study
pAE2567	pSLF173-LacZ-E537-GAC	This study
pAE2568	pSLF173-LacZ-E537-GAU	This study
pAE2678	pSLF173-LacZ-Y503-UGU	This study
pAE2679	pSLF173-LacZ- Y503-UGC	This study

Supplementary Table S3: Oligonucleotides used in this study

XhoI_LacZ_pJC27.fw	CGATCACTCGAGATGATTACGCCAAGCTTGGCTGC
LacZ_pJC27_BglII.rev	CGATCAAGATCTTTATTTTTGACACCAGACCAACTGG
Mm AspRTprimer	CTCAACTGGATTGGCTNNNNNGATAAATCCAGTTGAGTGGCTCCCCGTC
Mm Asp bisulfite.fw	GTTAGTATAGTGGTGAGTAT
Stemloop.rev	CTCAACTGGATTGGCT
His_Probe	AACCACTGGACCATGTGAGC
Asn_Probe	ACCAACTATGCTACCCGACC
Asp_Probe	TACCCCTATACTAAAGGAGA
Tyr_Probe	TCTCCTGAGCCAGAATCGAAC
Ser_AGA_Probe	GATTTCTAGTCTGTCCCTTT
5S_Probe	CGCCTAGGTATGGCCGTAGAC

Supplementary Table S4: Software used in this study

Software	Reference	Version
Bowtie	(11)	v1.1.2
Xtail	(12)	v1.1.5
R	www.R-project.org	v3.3.3
Python	www.python.org	v2.7.6
Galaxy	(13)	V1.1 locally installed

Supplementary Table S5: $tRNA^{Glu}$ methylation upon *pmt1⁺* overexpression

tRNA	m^5C38 in wt in EMM	p-value, m^5C38 in wt	m^5C38 in *pmt1*$^+$ OE	p-value, m^5C38 in *pmt1*$^+$ OE	p-value, differential
tRNA-Asp-GTC	0.1234	<2.2e-16	0.9476	<2.2e-16	<2.2e-16
tRNA-Glu-CTC	0.0159	1	0.0953	<2.2e-16	<2.2e-16
tRNA-Glu-TTC	0.0148	1	0.1332	<2.2e-16	<2.2e-16

Supplementary Table S6. Genes differentially translated in *pmt1*Δ + Q/ *pmt1*Δ (p<0.05)

Supplementary Table S7. Genes differentially translated in wt + Q/ *pmt1*Δ (padj. < 0.1)

Supplementary Table S8. Genes differentially translated in wt+Q/ wt (p < 0.05)

Supplementary Table S9. Genes differentially translated in *pmt1*$^+$ OE/ *pmt1*Δ + v (padj < 0.1)

Supplementary Table S10. Genes differentially translated in wt+Q/ *pmt1*Δ+Q (padj < 0.1)

For Supplementary Tables S6 – S10, see separate Excel file

Supplementary Table S11. Experimental conditions that have no effect on wt, *pmt1*Δ or *qtr2*Δ strains in the presence or absence of Q

Condition	Remark
Anisomycin	Translation inhibitor
Hygromycin	Translation inhibitor
Paromomycin	Translation inhibitor
Cycloheximide	Translation inhibitor
Rapamycin	Inhibitor of TOR signaling
UV irradiation	Mutagenic
Chronological aging	Conducted as in Stephan and Ehrenhofer-Murray (14)
Endogenous expression of colicin E5	Anticodon nuclease, cleaves the Q group of tRNAs (15)
Sensitivity to killer toxin from *K. lactis*	*S. pombe* was resistant to *K. lactis* killer strains
High temperature (37°)	Becker et al (9)
H_2O_2 sensitivity	Becker et al (9)
Nitrogen starvation	Becker et al (9)
NaCl sensitivity	Osmotic stress

Supplementary Table S12. Spearman rank correlations of codon A-site occupancy with the inverse of the RNA adaptation index (tAI) in different experiments.

Sample	Spearman rank correlation
wt	0.0679
wt + Q	0.0795
pmt1Δ	0.0929
pmt1Δ + Q	0.0940
pmt1Δ + v	0.1508
pmt1^{+} OE	0.0608

Supplementary materials and methods

Acryloyl aminophenylboronic acid (APB) gels –Northern blotting

APB gels were performed according to (16,17) with a few modifications. Brifely, 7 µg of RNA were deacetylated and re-suspended in 1x RNA loading dye (Fermentas). Samples were denatured for 10 min at 70 °C, loaded on APB gels, and run at 4 °C in 1 x TAE at 90 V for 30 min and 140 V for 3 hours. RNA was then transferred using a semi-dry system onto positively charged nylon membranes in 1X TAE at 5 V for 40 min, cross-linked with UV and sequentially hybridized as described previously (18).

^{35}S labeling of mitochondrial proteins

Radioactive labeling of mitochondrial translation products was carried out as described (19). Briefly, cells were grown in complete medium (YES, with or without Q) and harvested at early exponential phase. Cytosolic translation was blocked with 1mg/ml anisomycin for 20 min. ^{35}S labeled methionine/ cysteine (Met-35S-Label, Hartmann Analytic) was subsequently added, and cells were incubated shaking for 3 h. Proteins were extracted as in (20), separated by 17% SDS-PAGE and blotted onto nitrocellulose membranes. Membranes were exposed to a Phosphorimager screen at room temperature.

Mitochondrial membrane potential assay

Mitochondrial membrane potential was measured using the TMRE (tetramethylrhodamine ethyl ester)-Mitochondrial Membrane Potential Assay Kit (Abcam, ab113852) according to the manufacturer's instructions. Briefly, during early exponential phase of *S. pombe* cells, 1 µM TMRE was added to $7x10^5$ cells in complete medium (YES). After 30 min incubation at 30°C, TMRE fluorescence was measured using a BD Accuri C6 Flow Cytometer (BD Biosciences). 10^4 cells were analyzed in the FL2 cannel (585/40).

Literature:

1. Ingolia, N.T., Ghaemmaghami, S., Newman, J.R. and Weissman, J.S. (2009) Genome-wide analysis in vivo of translation with nucleotide resolution using ribosome profiling. *Science*, **324**, 218-223.

2. Brar, G.A., Yassour, M., Friedman, N., Regev, A., Ingolia, N.T. and Weissman, J.S. (2012) High-resolution view of the yeast meiotic program revealed by ribosome profiling. *Science*, **335**, 552-557.

3. Chou, H.J., Donnard, E., Gustafsson, H.T., Garber, M. and Rando, O.J. (2017) Transcriptome-wide Analysis of Roles for tRNA Modifications in Translational Regulation. *Mol Cell*, **68**, 978-992 e974.

4. Zinshteyn, B. and Gilbert, W.V. (2013) Loss of a conserved tRNA anticodon modification perturbs cellular signaling. *PLoS genetics*, **9**, e1003675.

5. Duncan, C.D.S. and Mata, J. (2017) Effects of cycloheximide on the interpretation of ribosome profiling experiments in Schizosaccharomyces pombe. *Scientific reports*, **7**, 10331.

6. Gerashchenko, M.V. and Gladyshev, V.N. (2014) Translation inhibitors cause abnormalities in ribosome profiling experiments. *Nucleic Acids Res*, **42**, e134.

7. Hussmann, J.A., Patchett, S., Johnson, A., Sawyer, S. and Press, W.H. (2015) Understanding Biases in Ribosome Profiling Experiments Reveals Signatures of Translation Dynamics in Yeast. *PLoS genetics*, **11**, e1005732.

8. Huang da, W., Sherman, B.T. and Lempicki, R.A. (2009) Systematic and integrative analysis of large gene lists using DAVID bioinformatics resources. *Nature protocols*, **4**, 44-57.

9. Becker, M., Muller, S., Nellen, W., Jurkowski, T.P., Jeltsch, A. and Ehrenhofer-Murray, A.E. (2012) Pmt1, a Dnmt2 homolog in Schizosaccharomyces pombe, mediates tRNA methylation in response to nutrient signaling. *Nucleic Acids Res*, **40**, 11648-11658.

10. Müller, M., Hartmann, M., Schuster, I., Bender, S., Thuring, K.L., Helm, M., Katze, J.R., Nellen, W., Lyko, F. and Ehrenhofer-Murray, A.E. (2015) Dynamic modulation of Dnmt2-dependent tRNA methylation by the micronutrient queuine. *Nucleic Acids Res*, **43**, 10952-10962.

11. Langmead, B., Trapnell, C., Pop, M. and Salzberg, S.L. (2009) Ultrafast and memory-efficient alignment of short DNA sequences to the human genome. *Genome biology*, **10**, R25.

12. Xiao, Z., Zou, Q., Liu, Y. and Yang, X. (2016) Genome-wide assessment of differential translations with ribosome profiling data. *Nature communications*, **7**, 11194.

13. Goecks, J., Nekrutenko, A., Taylor, J. and Galaxy, T. (2010) Galaxy: a comprehensive approach for supporting accessible, reproducible, and transparent computational research in the life sciences. *Genome biology*, **11**, R86.

14. Stephan, J. and Ehrenhofer-Murray, A.E. (2015) A method for high-throughput analysis of chronological aging in Schizosaccharomyces pombe. *Methods Mol Biol*, **1263**, 93-101.

15. Ogawa, T., Tomita, K., Ueda, T., Watanabe, K., Uozumi, T. and Masaki, H. (1999) A cytotoxic ribonuclease targeting specific transfer RNA anticodons. *Science*, **283**, 2097-2100.

16. Igloi, G.L. and Kossel, H. (1985) Affinity electrophoresis for monitoring terminal phosphorylation and the presence of queuosine in RNA. Application of polyacrylamide containing a covalently bound boronic acid. *Nucleic Acids Res*, **13**, 6881-6898.

17. Zaborske, J.M., DuMont, V.L., Wallace, E.W., Pan, T., Aquadro, C.F. and Drummond, D.A. (2014) A nutrient-driven tRNA modification alters translational fidelity and genome-wide protein coding across an animal genus. *PLoS Biol*, **12**, e1002015.

18. Tuorto, F., Liebers, R., Musch, T., Schaefer, M., Hofmann, S., Kellner, S., Frye, M., Helm, M., Stoecklin, G. and Lyko, F. (2012) RNA cytosine methylation by Dnmt2 and NSun2 promotes tRNA stability and protein synthesis. *Nature structural & molecular biology*, **19**, 900-905.

19. Kuhl, I., Dujeancourt, L., Gaisne, M., Herbert, C.J. and Bonnefoy, N. (2011) A genome wide study in fission yeast reveals nine PPR proteins that regulate mitochondrial gene expression. *Nucleic Acids Res*, **39**, 8029-8041.

20. Gouget, K., Verde, F. and Barrientos, A. (2008) In vivo labeling and analysis of mitochondrial translation products in budding and in fission yeasts. *Methods Mol Biol*, **457**, 113-124.
